# Global phylogeography and invasion history of the spotted lanternfly revealed by mitochondrial phylogenomics

**DOI:** 10.1111/eva.13170

**Published:** 2020-12-14

**Authors:** Zhenyong Du, Yunfei Wu, Zhuo Chen, Liangming Cao, Tadashi Ishikawa, Satoshi Kamitani, Teiji Sota, Fan Song, Li Tian, Wanzhi Cai, Hu Li

**Affiliations:** ^1^ Department of Entomology MOA Key Lab of Pest Monitoring and Green Management College of Plant Protection China Agricultural University Beijing China; ^2^ The Key Laboratory of Forest Protection National Forestry and Grassland Administration Research Institute of Forest Ecology, Environment and Protection Chinese Academy of Forestry Beijing China; ^3^ Laboratory of Entomology Faculty of Agriculture Tokyo University of Agriculture Atsugi Japan; ^4^ Entomological Laboratory Graduate School of Bioresource and Bioenvironmental Sciences Kyushu University Fukuoka Japan; ^5^ Department of Zoology Graduate School of Science Kyoto University Sakyo Japan

**Keywords:** invasion history, *Lycorma delicatula*, mitochondrial genome, phylogeography

## Abstract

Biological invasion has been a serious global threat due to increasing international trade and population movements. Tracking the source and route of invasive species and evaluating the genetic differences in their native regions have great significance for the effective monitoring and management, and further resolving the invasive mechanism. The spotted lanternfly *Lycorma delicatula* is native to China and invaded South Korea, Japan, and the United States during the last decade, causing severe damages to the fruits and timber industries. However, its global phylogeographic pattern and invasion history are not clearly understood. We applied high‐throughput sequencing to obtain 392 whole mitochondrial genome sequences from four countries to ascertain the origin, dispersal, and invasion history of the spotted lanternfly. Phylogenomic analyses revealed that the spotted lanternfly originated from southwestern China, diverged into six phylogeographic lineages, and experienced northward expansion across the Yangtze River in the late Pleistocene. South Korea populations were derived from multiple invasions from eastern China and Japan with two different genetic sources of northwestern (Loess Plateau) and eastern (East Plain) lineages in China, whereas the each of Japan and the United States had only one. The United States populations originated through single invasive event from South Korea, which served as a bridgehead of invasion. The environmental conditions, especially the distribution of host Ailanthus trees, and adaptability possibly account for the rapid spread of the spotted lanternfly in the native and introduced regions.

## 1 INTRODUCTION

Biological invasion represents one of the most serious biosafety issues all around the world, causing severe threats to public health, environments, and agriculture (Sax et al., 2005; Sileshi et al., 2019). Invasive pests can spread from its native regions primarily through human activities, such as international trade and travel (Kim et al., 2017; Leskey & Nielsen, 2018; Paini et al., 2016). However, effective managements are challenging due to the difficulty of tracking and predicting their spread routes. Explicit knowledge of invasion history inferred from molecular markers can promote effective monitoring and quarantine aimed at the source regions (Ascunce & Shoemaker, 2011).

Defining the accurate phylogeographic pattern and demographic history of native and invasive populations is crucial for effective management of invasive pests, because different control measures need to be applied to those populations with significant genetic structures and different sources. Studying genetic diversity of native and invasive pest populations can also provide important insights understanding the successful invasion process. For instance, higher genetic diversity might be associated with stronger adaptive abilities (Garnas et al., 2016), and low genetic diversity among invasive population may suggest the presence of bottleneck or founder effects (small initial invasive population) during initial invasion (Javal et al., 2019; Szűcs, Melbourne, et al., 2017). In addition, comparing genetic background of native and different invasive populations may inform the sites of secondary introductions and intermediate invasion regions between native and an introduced country (Bertelsmeier & Keller, 2018; Qiao et al., 2019). A comprehensive understanding of genetic diversity and invasion history of invasive pests is fundamental for future study on the genomic mechanism behind their successful invasions.

The spotted lanternfly (SLF), *Lycorma delicatula* (White), is a phytophagous hemipteran insect first recorded and commonly found in northern China (Liu, 1939). In China, this hopper is not regarded as a serious pest because it primarily feeds on non‐economic plants, including *Ailanthus altissima* (Miller) Swingle (tree‐of‐heaven), *Melia azedarach* Linnaeus and *Vitis* plants. However, in its new habitats, it feeds on grapevines and other fruit trees, leading to a huge decline in the quality and yield of grapes and causing severe economic losses to the fruits industry. The invasion of the SLF into other country was first confirmed in 2004, when it was found in western South Korea. This is followed by its rapid spread across the country (Han et al., 2008). Subsequently, it was identified in Ishikawa Prefecture, Japan (Kim et al., 2013; Tomisawa et al., 2013), and recently in the eastern United States, including 13 counties in southeastern Pennsylvania and nine other surrounding states (NYSIPM, 2020), despite that persistent quarantine efforts surrounding the first detection site have been made. Because the SLF is the only fulgorid planthopper in northeastern North America, very few natural enemies have been reported attacking it, so that the spread of this hopper will likely continue and increase further (Clifton et al., 2019). Commercial transportation of more than 70 fruit and timber plants in 25 families, which are potential hosts, may be involved in its rapid, worldwide spread (Dara et al., 2015). For these phytophagous insects, the environmental conditions, especially food resources, play a pivotal role in determining successful colonization and spread in the introduced regions. Adaptive evolution sometimes acts as an important factor influencing the invasive process (Szűcs, Vahsen, et al., 2017; Wu et al., 2019).

Several phylogeographic and population genetic studies have partially revealed the invasion process of the SLF between East Asian countries (Kim et al., 2013; Park et al., 2013; Zhang et al., 2019). Analysis of specimens from China, South Korea, and Japan suggest that native populations in northern China of the SLF is likely the source of introduction to Japan and South Korea (Kim et al., 2013; Zhang et al., 2019), and that multiple independent invasion in the western coastal regions, followed by long‐distance, inland dispersal may account for the rapid spread of the SLF throughout South Korea (Park et al., 2013). It remains unknown, however, how this hopper crosses the Pacific Ocean, arrives and spreads in the United States (Kim et al., 2013; Park et al., 2013; Zhang et al., 2019).

Previous phylogeographic analysis for SLF, based on limited population sampling and a few mitochondrial markers have been unable to reveal detailed invasion history due to limited phylogenetic resolutions (Hirase et al., 2016). Mitochondrial genome (mitogenome) is a complete organelle genome in eukaryotic cells, comprising 37 coding genes and a control region (Cameron, 2014). Mitochondrial genes have short coalescent times, unambiguous orthology, and rapid evolutionary rates compared to nuclear genes (Allio et al., 2017) and have been the foundation of phylogeography studies for the past 30 years (Hickerson et al., 2010). Recently, whole mitogenome sequences have been used in animal phylogeography to provide high resolution phylogenetic tree (Bjork et al., 2011; Chang et al., 2017; Du et al., 2020; Fields et al., 2018; Ma et al., 2012; Morin et al., 2010). The development of high‐throughput sequencing offers a cost‐effective approach to study large‐scale mitogenomic phylogeography, especially for recently diverged species (Du et al., 2019). To obtain a comprehensive understanding on phylogeographic pattern and invasion history from East Asia to North America, we analyzed whole mitogenome sequences of 392 individuals, representing a total of 40 native and invasive populations. We estimated divergence time and demographic history of different phylogeographic lineages of the SLF. Based on these results, we discussed the origin, dispersal, and demographic history of this hopper in China, as well as its spread route into other parts of the world, including North America.

## 2 MATERIALS AND METHODS

### 2.1 Sampling and total genomic DNA extraction

**FIGURE 1:**
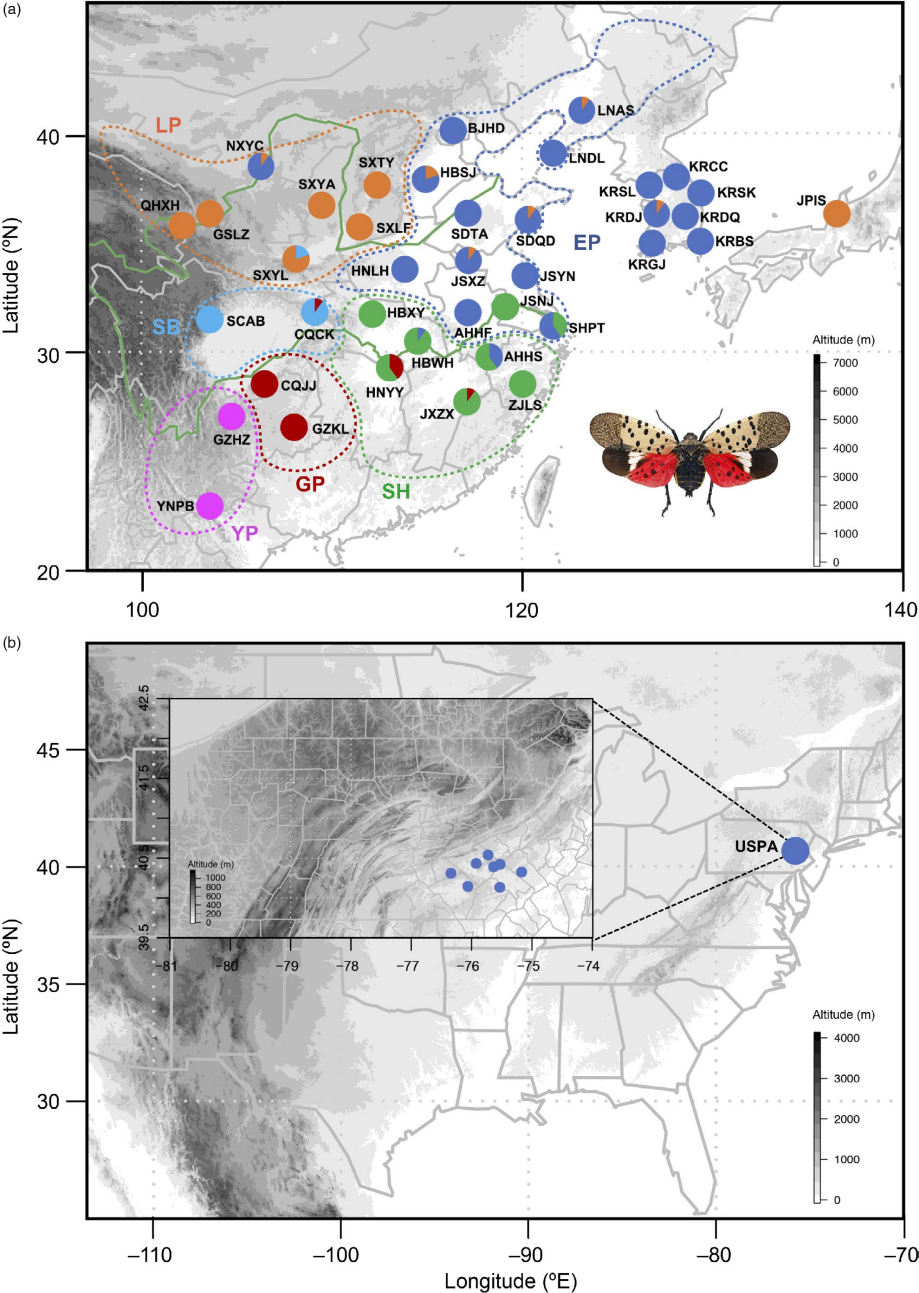
Geographical distribution of 40 sample populations in East Asia and United States. The colors of the circles represent the six phylogeographic lineages referring to Figure 3b, and the area of the circle is proportional to the number of individuals except YNPB. See Table 1 for population codes. The boundaries of six topographical areas are shown with dashed lines

A total of 392 SLF specimens were collected from 40 populations in China, South Korea, Japan, and the United States, covering the native and invasive regions of this species (Figure 1, Table 1). One specimen of *Lycorma meliae* collected in New Taipei City of Taiwan was included as an outgroup. All samples were stored immediately in absolute ethanol in the field and transferred to −80°C freezer back to the laboratory. Total genomic DNA was extracted from the muscles of thorax using a DNeasy Blood & Tissue Kit (QIAGEN).

**TABLE 1:** Sample localities and genetic diversity of spotted lanternfly

Population	Sample locality	Code	Latitude	Longitude	Date	*N*	*S*	Nh	Hd	*π*
**China**						302	731	212	0.9931	0.00687
1	Shibei District, Qingdao, Shandong	SDQD1–10	36.1206	120.3690	September 2017	10	21	5	0.844	0.00043
2	Taishan District, Taian, Shandong	SDTA1–10	36.1986	117.1131	September 2017	10	21	8	0.933	0.00048
3	Haidian District, Beijing	BJHD1–10	40.0253	116.2828	August 2017	10	23	9	0.978	0.00035
4	Jinyuan District, Taiyuan, Shanxi	SXTY1–10	37.6661	112.4249	September 2017	10	16	8	0.956	0.00028
5	Qvwo Country, Linfen, Shanxi	SXLF1–10	35.6569	111.4757	July 2018	10	19	8	0.956	0.00037
6	Yancheng District, Luohe, Henan	HNLH1–10	33.7066	113.7939	September 2017	10	18	9	0.978	0.00029
7	Yangling, Xianyang, Shannxi	SXYL1–10	34.2623	108.0737	August 2017	10	40	10	1	0.00092
8	Baota District, Yan'an, Shannxi	SXYA1–10	36.6190	109.4571	July 2017	10	27	9	0.978	0.00061
9	Yuhua District, Shijiazhuang, Hebei	HBSJ1–10	37.9757	114.5153	September 2017	10	24	7	0.933	0.00055
10	Putuo District, Shanghai	SHPT1–10	31.2323	121.4692	July 2017	10	196	10	1	0.00689
11	Honggu District, Lanzhou, Gansu	GSLZ1–10	36.1679	103.2568	June 2017	10	16	5	0.677	0.00039
12	Xunhua County, Haidong, Qinghai	QHXH1–10	35.8514	102.4873	July 2017	10	7	4	0.644	0.00017
13	Zhongshan District, Dalian, Liaoning	LNDL1–10	38.9109	121.6529	August 2017	10	7	5	0.844	0.00017
14	Tiedong District, Anshan, Liaoning	LNAS1–10	41.1044	123.0080	September 2017	10	22	6	0.844	0.00041
15	Shushan District, Hefei, Anhui	AHHF1–10	31.7762	117.1804	September 2017	10	9	7	0.911	0.0002
16	Huizhou District, Huangshan, Anhui	AHHS1–10	29.8159	118.2682	July 2017	10	206	9	0.978	0.00713
17	Yueyanglou District, Yueyang, Hunan	HNYY1–10	29.3528	113.1655	August 2017	10	108	8	0.933	0.00304
18	Dongxihu District, Wuhan, Hubei	HBWH1–10	30.6426	114.1818	August 2017	10	193	9	0.978	0.0028
19	Xiangcheng District, Xiangyang, Hubei	HBXY1–10	32.0106	112.1743	August 2017	10	38	9	0.978	0.0006
20	Hezhang Country, Bijie, Guizhou	GZHZ1–10	27.1307	104.7313	August 2017	10	47	8	0.956	0.00131
21	Kaili City, Qiandongnan, Guizhou	GZKL1–10	26.5808	107.9831	September 2017	10	24	6	0.911	0.00061
22	Zixi Country, Fuzhou, Jiangxi	JXZX1–10	27.7206	117.0832	August 2017	10	91	9	0.978	0.00178
23	Liandu District, Lishui, Zhejiang	ZJLS1–10	28.4473	119.9728	August 2017	10	75	8	0.956	0.00126
24	Tongshan District, Xuzhou, Jiangsu	JSXZ1–10	34.1996	117.1785	July 2017	10	25	7	0.867	0.00037
25	Tinghu District, Yancheng, Jiangsu	JSYN1–10	33.3840	120.1687	August 2017	10	0	1	0	0
26	Xuanwu District, Nanjing, Jiangsu	JSNJ1–10	32.0559	118.8242	August 2017	10	21	7	0.911	0.00053
27	Xixia District, Yinchuan, Ningxia	NXYC1–10	38.5359	106.1324	August 2017	10	23	7	0.911	0.00041
28	Jiangjin District, Chongqing	CQJJ1–10	28.6017	106.3381	August 2017	10	33	8	0.933	0.00066
29	Chengkou District, Chongqing	CQCK1–10	31.7711	109.0956	August 2017	10	202	10	1	0.00292
30	Li Country, Aba, Sichuan	SCAB1–10	31.4903	103.2094	September 2017	10	13	5	0.756	0.00031
31	Pingbian Country, Honghe, Yunnan	YNPB1–2	22.9863	103.6751	August 2017	2	9	2	1	0.00063
**Pennsylvania State, USA**						10	0	1	0	0
32	Kutztown Rd, Berks	USPA1	40.5380	–75.7267	June 2017	1	0	1	0	0
	Anthonys Mill Rd, Berks	USPA2–3	40.3902	–75.6334	July 2017	2	0	1	0	0
	Ontelaunee Township, Berks	USPA4	40.4325	–75.9235	August 2017	1	0	1	0	0
	647 Nutt Road, Chester	USPA5	40.1336	–75.5319	August 2017	1	0	1	0	0
	2208 S 5th Ave, Lebanon	USPA6	40.3098	–76.3416	September 2017	1	0	1	0	0
	Peace Valley Park, Bucks	USPA7	40.3246	–75.1697	September 2017	1	0	1	0	0
	Palm Hill Road, Montgomery	USPA8–9	40.4212	–75.5285	September 2017	2	0	1	0	0
	Blue Rock Rd, Lancaster	USPA10	40.1449	–76.0615	September 2017	1	0	1	0	0
**South Korea**						70	59	31	0.916	0.00025
33	Hoegi‐dong, Dongdaemun‐gu, Seoul	KRSL1–10	37.5962	127.0504	August 2018	10	10	5	0.756	0.00019
34	Gyo‐dong, Samcheog, Gangwon‐do	KRSK1–10	37.4539	129.1579	August 2018	10	4	4	0.644	0.00009
35	Yongbong‐dong, Buk‐gu, Kwangju	KRGJ1–10	35.1761	126.9036	August 2018	10	5	6	0.778	0.0001
36	Jangjeon‐dong, Geumjeong‐gu, Busan	KRBS1–10	35.2341	129.0760	August 2018	10	10	5	0.667	0.00021
37	Gung‐dong, Yuseong‐gu, Daejeon	KRDJ1–10	36.3626	127.3415	August 2018	10	26	8	0.933	0.00038
38	Seoksa‐dong, Chuncheon, Gangwon‐do	KRCC1–10	37.8695	127.7423	August 2018	10	9	7	0.911	0.0002
39	Daehyeon‐dong, Buk‐gu, Daegu	KRDQ1–10	35.8901	128.6113	August 2018	10	16	7	0.867	0.00038
**Japan**						10	11	5	0.822	0.00021
40	Futsumachi, Komatsu, Ishikawa, Japan	JPIS1–10	36.3551	136.4172	September 2017	10	11	5	0.822	0.00021
**All**						392	755	244	0.9892	0.00583

Abbreviations: Hd, haplotype diversity; *N*, sample size; Nh, number of haplotypes; *S*, number of segregating sites; *π*, nucleotide diversity.

### 2.2 Mitochondrial metagenomic sequencing and assembly from pooled DNA

DNA from each SLF specimen was pooled with equimolar quantities of genomic DNA from another two species studied in our laboratory (a lace bug and an assassin bug) to reduce the sequencing expense after several preliminary experiments (Gillett et al., 2014; Hahn et al., 2013). A library with insert size of 350 bp was constructed from each sequencing pool and 150 bp paired end reads were sequenced to acquire at least 6 Gb data through Illumina NovaSeq 6000 platform (Berry Genomics). Clean reads were obtained after trimming adapters using Trimmomatic (Bolger et al., 2014). The published mitogenome (GenBank accession EU909203) was used as a reference in our study. For each library, clean reads were mapped onto the reference mitogenome (with the sequencing coverage summarized in Table S6), using Geneious 11.1.4 (http://www.geneious.com/) with parameters of up to 5% mismatches, a maximum gap size of 5 bp, and minimum overlap of 40 bp with at least 95% similarity. Due to the distant relationships of the three species and the strict similarity and continuity requirements of the parameters, only reads of SLF were allowed to be isolated and mapped onto the reference genome from the sequencing readpool. The consensus sequences were generated as novel individual mitogenomes under threshold of 75% uniformity each site. The mitogenomic assembly of *L. meliae* also followed this workflow with additional mapping iterations to fish reads to known regions until the whole sequence was constructed (Hahn et al., 2013). Annotation of transfer RNA (tRNA) genes was rechecked using MITOS Web Server (Bernt et al., 2013). Protein‐coding genes (PCGs) and ribosomal RNA (rRNA) genes were aligned with homologous genes of the reference mitogenome.

### 2.3 Phylogenetic analysis and network

Twenty‐two tRNAs, two rRNAs, one control region, and several non‐coding regions of the mitogenome were aligned respectively using MAFFT 7.0 online server with the G‐INS‐i strategy (Katoh & Standley, 2013). Thirteen PCGs were also aligned using TranslatorX online platform (Abascal et al., 2010) with all stop codons removed. In this study, we prepared two concatenated datasets in the following analysis: (a) the WG dataset, which contained the complete mitogenome sequences; and (b) the PRT dataset, which included all 13 PCGs, rRNA, and tRNA sequences. Bayesian analysis of population structure was performed using BAPS 6.0 (Cheng et al., 2013), based on the WG and PRT datasets under the spatial clustering of groups of individuals.

Phylogenetic reconstruction was performed based on the haplotypes of WG dataset. Haplotype data were generated using DnaSP 6.0 (Rozas et al., 2017). PartitionFinder2 (Lanfear et al., 2012) was used to select the best‐fit schemes and substitution models with “greedy” algorithm and Akaike information criterion. Although 44 partitions were predefined for the WG dataset, including 39 codon partitions for PCGs, two for rRNA, and three for tRNA, control region, and non‐coding region. A single scheme with GTR + I + G model was selected ultimately. Maximum‐likelihood (ML) analysis was applied with 1,000 replicates using ultrafast bootstrap approximation approach (Minh et al., 2013) in IQ‐TREE 1.6.5 (Trifinopoulos et al., 2016). Bayesian inference (BI) analysis was performed using MrBayes 3.2.2 (Ronquist & Huelsenbeck, 2003) with two simultaneous Markov chain Monte Carlo (MCMC) runs of 2 million generations. All trees were sampled every 1,000 generations, and first 25% of them were discarded as burn‐in.

Median‐joining networks (Bandelt et al., 1999) were constructed separately for three lineages resolved in phylogenetic analysis above based on the PRT dataset using PopART (population analysis with reticulate trees) (Leigh & Bryant, 2015). Haplotype frequency was calculated using Arlequin 3.5 (Excoffier & Lischer, 2010).

### 2.4 Genetic diversity and population differentiation

To test genetic diversity divergence across geographic populations and phylogenetic lineages in our study, the number of polymorphic sites (*S*), the number of haplotypes (*H*), haplotype diversity (Hd), and nucleotide diversity (*π*) were calculated based on the PRT dataset using DnaSP software. We used Arlequin software to calculate the genetic differentiation *F*_ST_ values and to obtain *p*‐values for *F*_ST_ values using 3,000 permutations. MEGA7 software (Kumar et al., 2016) was used to calculate uncorrected *p*‐distance between different populations, lineages, and species to check their differentiation levels. Mantel tests were performed to test the isolation‐by‐distance model for all populations. *F*_ST_ and linear geographic distance (ln km) were plotted with 999 replicates in the ade4 v. 1.7 module of R with the introduced populations excluded.

### 2.5 Divergence time estimation

Divergence time was calculated using BEAST 2.4.8 (Bouckaert et al., 2014) based on the PRT dataset. Considering there are no fossils available for the calibration of this species, we used a molecular clock for divergence time estimation. In addition, as all gene sequences were estimated as a single scheme with GTR + I + G model above, *cox1* gene was evaluated as a separate partition with the substitution rate fixed at 1.77% per site per million years (Papadopoulou et al., 2010). Other PCGs, rRNA and tRNA genes were applied as another three partitions, of which the evolutionary rates were estimated accordingly. An uncorrelated lognormal relaxed clock model was applied with the birth‐death model for the tree prior. Two independent MCMC runs were performed for 500 million generations with tree sampling every 50,000 generations. We used Tracer 1.7 (Rambaut et al., 2018) to verify that the MCMC runs had reached a stationary distribution based on the effective sample sizes (ESS) of each estimated parameter to be larger than 200. TreeAnnotator program in BEAST 2 software was used to generate the consensus tree with “mean height” after discarding the first 25% trees as burn‐in.

### 2.6 Demographic history

The neutral tests of Tajima's *D* (Tajima, 1989) and Fu's Fs (Fu, 1997) were performed using Arlequin software with default parameter settings. Tajima's *D* compares the number of segregating sites to the average number of pairwise differences, and Fu's Fs is based on the probability to observe a certain number of alleles given the average number of pairwise differences. The *p*‐values of these two statistics were obtained by comparing the observed statistics values with 1,000 simulated values under the hypothesis of selective neutrality and population equilibrium (Excoffier & Lischer, 2015). The mismatch distribution analyses were also performed for the six lineages using Arlequin software with default parameter settings. The mismatch distribution analysis computes the distribution of the observed number of differences between pairs of haplotypes in the populations, which is usually multimodal in demographic equilibrium population but unimodal in recent expansion populations (Rogers & Harpending, 1992). The departure of the observed mismatch distribution from the expected distribution under the expansion hypothesis was tested by comparing the observed distribution with a simulated mismatch distribution resulting from a bootstrap analysis with 1,000 replicates; two statistics, the sum of square deviations (*SSD*) and Harpending's raggedness index (*r*) were used to study the difference between observed and expected distributions (Excoffier & Lischer, 2015). BSP analyses were applied for different lineages using Coalescent Bayesian Skyline model for the tree prior in BEAST 2 software. The same partitions and substitution rates were applied consistent with these in the estimation of divergence time. Two independent MCMC runs were conducted for 100 million generations sampling every 1,000 generations. Effective population size through time was determined using Tracer software to generate the plots.

## 3 RESULTS

### 3.1 Mitogenomic diversity among populations

A total of 392 whole mitogenome sequences with 37 functional coding genes and one control region were obtained from 40 populations worldwide (Figure 1, Table 1). The length of sequences varied from 15,915 to 15,931 bp, with 23.3%–23.4% GC contents. No large variations in gene arrangement or non‐coding regions were detected in different individuals of populations.

Based on the PRT dataset (including all PCGs, rRNA and tRNA genes), a total of 244 haplotypes were defined from 755 segregating sites. The overall Hd was 0.9892 and *π* for all populations was 0.00583. For these four sampling countries, populations of China exhibited the highest level of genetic diversity (Hd = 0.9931, *π* = 0.00687). The introduced populations of South Korea (Hd = 0.916, *π* = 0.00025) and Japan (Hd = 0.822, *π* = 0.00021) showed much lower diversity (Table 1). A single haplotype was detected in the United States with no polymorphic sites. Among all populations, Yangtze riverside populations in China like SHPT and AHHS exhibited the highest level of genetic diversity (Table 1, Figure 1).

The *F*_ST_ and genetic distances (*p*‐distances) between 40 populations were −0.0499 to 1 and 0.0001 to 0.0183 respectively, which were lowest between and within populations of eastern China and South Korea. Populations in different topographical areas, especially those separated by Yangtze River, had larger values (Tables S1 and S2). The Mantel test produced a significant correlation (*p* < .01) between the genetic and geographical distance in SLF populations (Figure S1).

### 3.2 Phylogeographic subdivisions and divergence time

**FIGURE 2:**
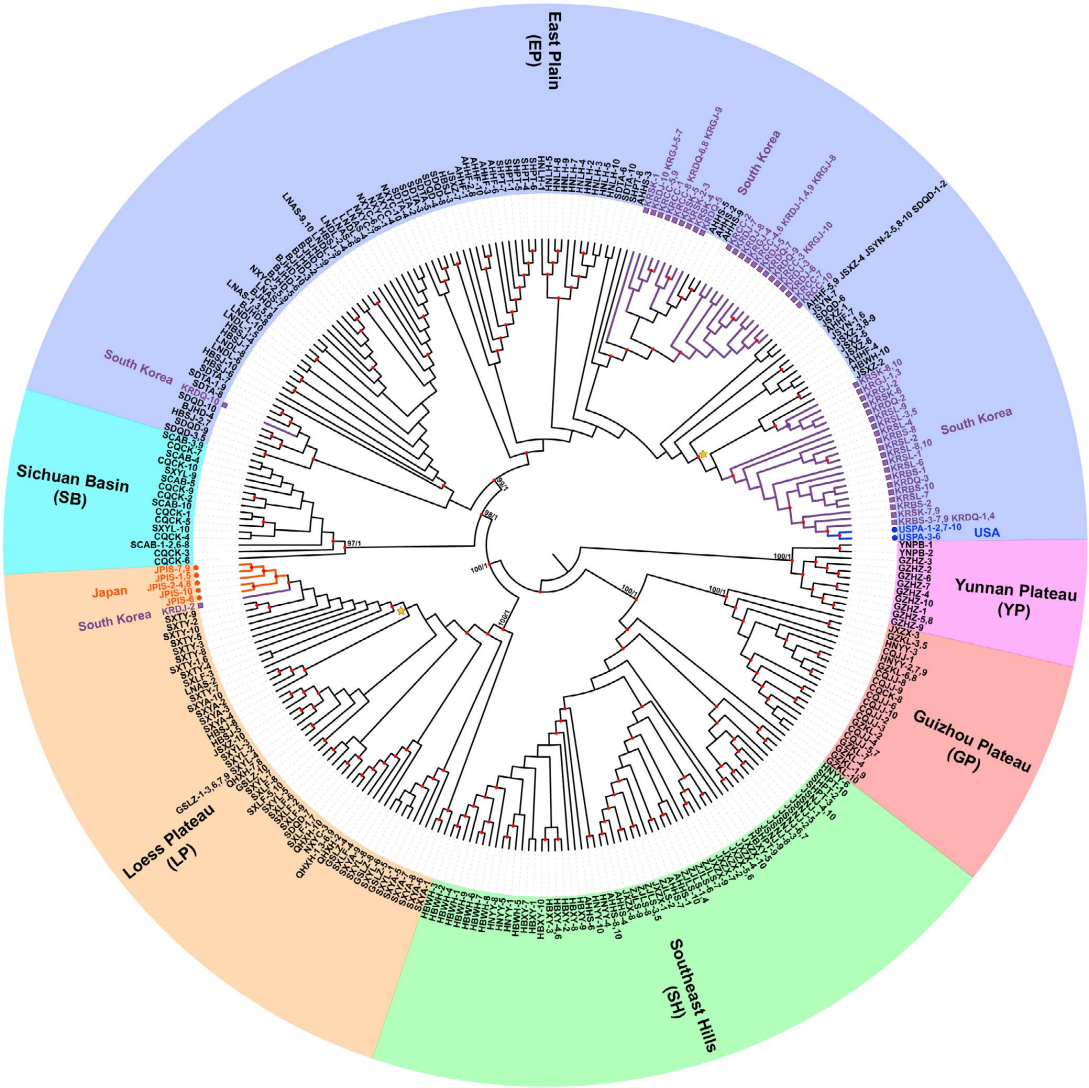
Phylogenetic topology based on mitogenomic haplotypes. The taxa labels describe all individuals sharing haplotypes. The nodal supports of major branches are bootstrap percentages and posterior probabilities. Red circles represent the nodes with bootstrap values larger than 70. The phylogeographic lineages and introduced countries are also shown. The branches with invasive haplotypes are labeled with different colors and geometric figures. The phylogenetic tree with branch length and detailed clades referred by two yellow stars are shown in Figure S2

**FIGURE 3:**
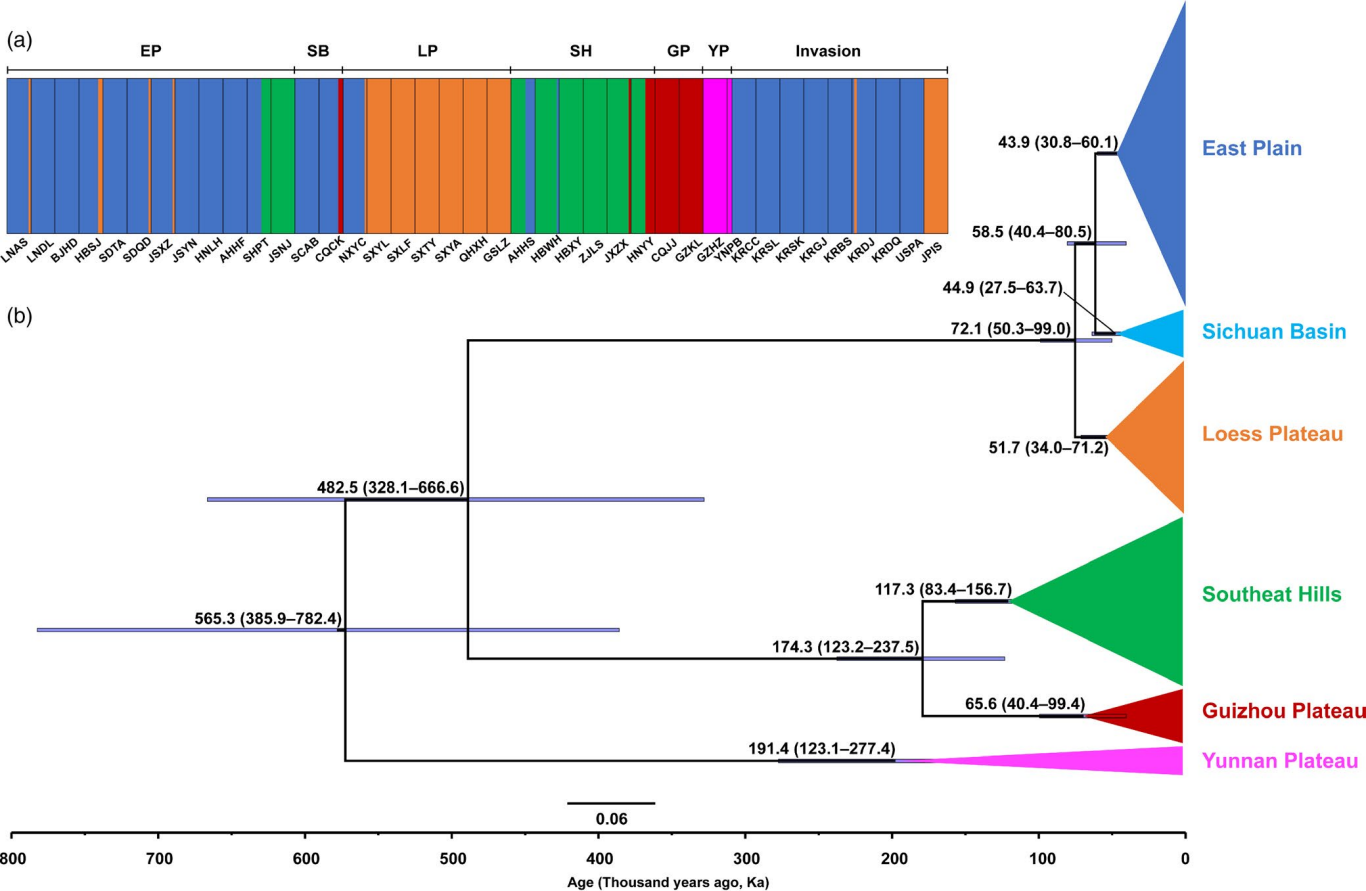
Clustering of individuals of 40 populations and estimated divergence time of the six phylogeographic lineages. (a) Different colors represent the five BAPS (Bayesian analysis of population structure)‐divided groups, with the EP (East Plain), and SB (Sichuan Basin) lineages clustered together. The populations were partitioned into six regions (see Figure 1) with the divisions indicated. (b) The values show the mean estimated time with 95% highest posterior density intervals in the brackets, as indicated by purple bars

The phylogenetic trees based on the WG dataset (including complete mitogenome sequence) using ML and BI methods contained six phylogeographic lineages (Figure 2). The distributions of the six lineages in China were geographically continuous and accordant with the important topographical areas in China. We therefore assigned the names of the lineages according to their topographical regions (Figure 1), including East Plain (EP), Loess Plateau (LP), Sichuan Basin (SB), Southeast Hills (SH), Guizhou Plateau (GP), and Yunnan Plateau (YP) lineages. Among these lineages, East Plain, Loess Plain, and Sichuan Basin are located in the northern Yangtze regions and the other three are in southern Yangtze regions. The (((EP, SB), LP), (SH, GP), YP) topology was resolved with high nodal support (Figures 2 and 3b). BAPS (Bayesian analysis of population structure) revealed a similar structure and all individuals were divided into five groups, because East Plain and Sichuan Basin lineages were included in one group (Figure 3a). The populations in the contact zones of different groups partially exhibited as populations with mixed haplotype lineages (Figure 1).

For the six lineages resolved in the phylogenetic analyses, the three lineages from southern Yangtze regions (Yunnan Plateau, Guizhou Plateau, and Southeast Hills) had higher diversity than those from northern Yangtze regions (Table 2). Yunnan Plateau lineage with the fewest individuals showed the highest *π* values, followed by Southeast Hills and Guizhou Plateau lineages (Table 2). For the three northern Yangtze lineages (East Plain, Sichuan Basin, and Loess Plateau), East Plain lineage exhibited the lowest diversity, even though it comprised the largest sample size (Table 2).

**TABLE 2:** Genetic diversity, neutrality test, and statistics of mismatch distribution of the six phylogeographic lineages

Group	*N*	*S*	Nh	Hd	*π*	Tajima's *D*	Fu's Fs	*SSD*	*r*
All samples	392	755	244	0.9892	0.00583	−0.96734	−24.02892*	0.01217	0.0025
East Plain (EP)	194	151	97	0.961	0.00038	−2.48542*	−24.86287*	0.00561	0.02393
Loess Plateau (LP)	75	93	49	0.978	0.00049	−2.19360*	−24.94783*	0.00429	0.00944
Sichuan Basin (SB)	21	49	16	0.948	0.00047	−2.03183*	−5.26722*	0.00929	0.02583
Southeast Hills (SH)	64	224	54	0.994	0.00156	−1.88471*	−21.58018*	0.00798	0.00485
Guizhou Plateau (GP)	26	75	18	0.969	0.00082	−1.57970	−1.80824	0.00511	0.00604
Yunnan Plateau (YP)	12	112	10	0.97	0.00255	−0.13734	1.67171	0.05017	0.05142

Abbreviations: Hd, haplotype diversity; *N*, sample size; Nh, number of haplotypes; *r*, Harpending's raggedness index; *S*, number of segregating sites; *SSD*, sum of square deviations; *π*, nucleotide diversity.

* *p* < .05.

*F*_ST_ among the six lineages varied from 0.5275 to 0.9677. The largest value was detected between East Plain and Yunnan Plateau lineages with the smallest variation between East Plain and Sichuan Basin lineages (above diagonal in Table 3). The genetic distance (uncorrected *p*‐distance) varied from 0.00085 to 0.01737 between the different lineages, being smallest between East Plain and Sichuan Basin lineages and largest between Southeast Hills and Yunnan Plateau lineages, which were both lower than 0.06021–0.06229 between two species (below diagonal in Table 3).

**TABLE 3:** Genetic distance (uncorrected *p*‐distance; below diagonal) and pairwise *F*_ST_ values (above diagonal) between lineages and species

Group	EP	LP	SB	SH	GP	YP
East Plain (EP)	0	0.65323	0.52746	0.94674	0.9661	0.96767
Loess Plateau (LP)	0.00120	0	0.61405	0.92477	0.95657	0.95181
Sichuan Basin (SB)	0.00085	0.00124	0	0.90144	0.95045	0.92307
Southeast Hills (SH)	0.01271	0.01293	0.01277	0	0.67058	0.90132
Guizhou Plateau (GP)	0.01238	0.01261	0.01243	0.00387	0	0.92214
Yunnan Plateau (YP)	0.01595	0.01607	0.01574	0.01737	0.01675	0
Outgroup (*L. meliae*)	0.06189	0.06196	0.06179	0.06096	0.06021	0.06229

In the phylogenetic trees, the United States and most South Korea haplotypes belonged to East Plain lineage, except one individual was classified into Loess Plateau lineage (Figure 2). All Japan haplotypes formed a monophyly group in Loess Plateau lineage, which was related to this single South Korea individual (Figure 2). The United States haplotypes had the closest relationship with some of the South Korea haplotypes. However, South Korea haplotypes were not monophyletic and divided into three different clades, each of which was close to haplotypes from eastern China populations (Figure 2).

The divergence time estimation suggested the most recent common ancestor (MRCA) of SLF diverged into Yunnan Plateau and other lineages with the MRCA age of 191.4 ka (kilo‐annum) and 482.5 ka. The other lineages then diverged into two clades located in southern and northern Yangtze regions (Figure 3b). The southern Yangtze clade diverged into Southeast Hills and Guizhou Plateau lineages 174.3 ka with MRCA age of 117.3 and 65.6 ka. The northern Yangtze clade diverged recently (MRCA age, 72.1 ka) into Loess Plateau (MRCA age, 51.7 ka) and East Plain and Sichuan Basin lineages (MRCA age, 58.5 ka). The MRCA ages of East Plain and Sichuan Basin lineages were similar at 43.9 and 44.9 ka, respectively (Figure 3b).

### 3.3 Haplotype networks

**FIGURE 4:**
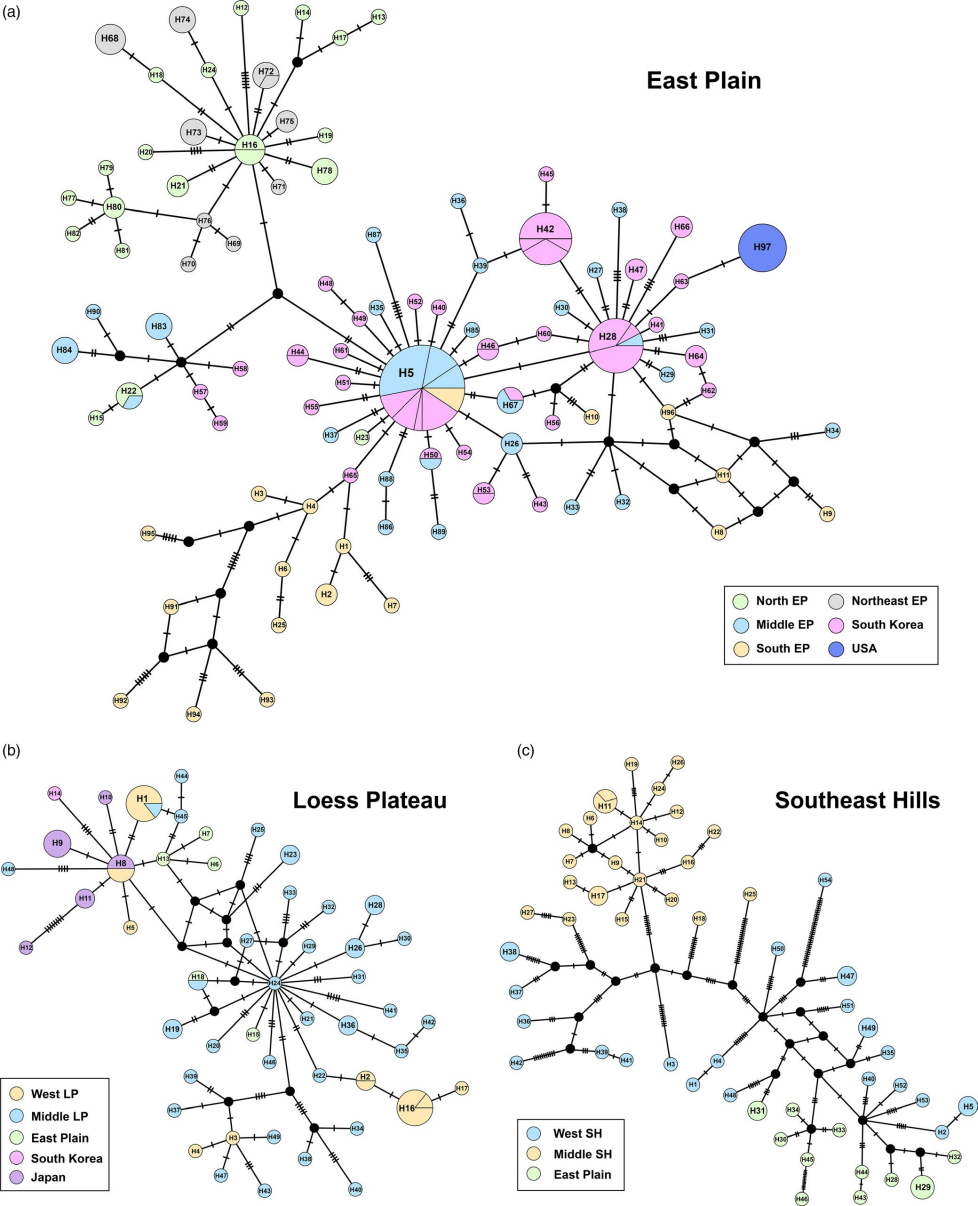
Median‐joining networks for three phylogeographic lineages. Colored circles represent different haplotypes, black circles represent missing haplotypes that were not observed, and solid lines between haplotypes represent mutation steps. The area of the circle is proportional to the number of haplotypes. See Tables S3–S5 for haplotype frequencies

The median‐joining networks supported similar results of the phylogenetic relationships. For the East Plain (EP) lineage, several typical star‐like topologies were identified for 22 populations, indicating the recent expansion in this lineage (Figure 4a). The H5 was the largest haplotype connected with H28 by one mutation. These two haplotype groups were composed of almost all South Korea haplotypes and partial China haplotypes from middle EP (HNLH, SDTA, SDQD, JSXZ, JSYN) and south EP (AHHS, AHHF, SHPT, HBWH). The United States haplotype had the closest relationship with one South Korea haplotype (H63) through one mutation step (Figure 4a). This haplotype was further connected with H28 by one mutation step, which contained South Korea and middle EP population. Apart from the two main haplotype groups, the northeast EP populations (LNAS, LNDL) were close to the north EP populations (BJHD, HBSJ, NXYC). The other haplotypes of south EP were distributed far from the main haplotype groups with relatively large mutations (Figure 4a; Table S3).

For the Loess Plateau (LP) lineage consisting of 13 populations, two central haplotypes were found with small sample size. H24 was connected with most haplotypes from middle LP (SXLF, SXTY, SXYA, SXYL) and two East Plain (EP) populations (LNAS, SDQD) by less than five mutations (Figure 4b). Only one South Korea haplotype (H14) had the closest relationship with H8 by four mutation steps. The five Japan haplotypes (H8–H12) were connected with the west LP inland China populations (GSLZ, QHXH) and other haplotypes of EP (HBSJ, JSXZ) with one to two mutations (Figure 4b; Table S4).

For the Southeast Hills (SH) lineage including eight populations, no shared haplotype was found among different populations except H11 (Figure 4c). Unlike the above two networks, there was no obvious central haplotype. The mutation steps were also larger than those in the other two lineages. Geographically adjacent haplotypes from west SH (HBWH, HBXY, HNYY), middle SH (AHHS, JXZX, ZJLS), and East Plain (EP; JSNJ, SHPT) were clustered closer through several missing haplotypes (Figure 4c; Table S5).

### 3.4 Demographic history

**FIGURE 5:**
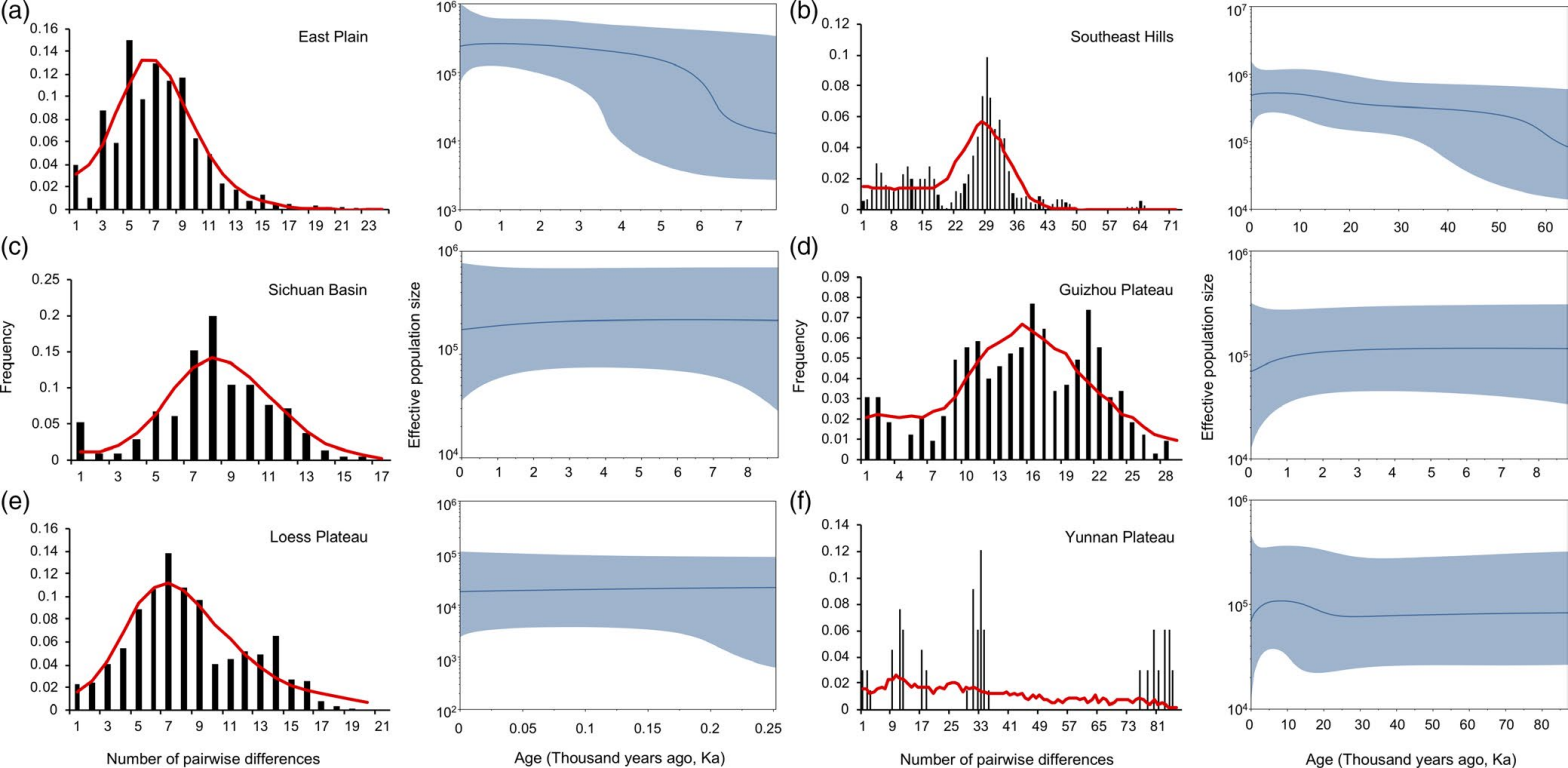
Mismatch distributions and Bayesian skyline plots for the six phylogeographic lineages. The vertical bars represent the observed mismatch distribution, and the red lines represent the expected distribution under the expansion model. For Bayesian skyline plots, the mean estimated effective population sizes (lines) are enclosed within the 95% highest posterior density intervals (shaded areas)

Multiple methods including neutral test, mismatch distribution, and Bayesian skyline plot (BSP) analyses were used to explore the demographic history of the six lineages. Significant negative values (*p* < .05) of Tajima's *D* and Fu's Fs were detected in four lineages, East Plain, Loess Plateau, Sichuan Basin, and Southeast Hills lineages (Table 2), indicating that population expansion occurred in these lineages. The Guizhou Plateau and Yunnan Plateau lineages did not show significant departure from zero (*p* > .05). In the mismatch distribution analysis, the observed and simulated curves did not significantly differ from each other in all lineages with the parameters of *SSD* and *r* (Table 2) being small and insignificant (*p* > .05). Hence, the population expansion process was not rejected in any of the lineages. In the BSP analyses, the trend of population expansion was only shown in the East Plain and Southeast Hills lineages (Figure 5a,b). The expansion of Southeast Hills lineage had begun at about 65 ka and East Plain lineage at 6–8 ka. The mean effective population sizes of the other lineages were stable through time (Figure 5c–f).

## 4 DISCUSSION

### 4.1 Phylogeographic pattern in native regions

Several studies have attempted to reveal the authentic phylogeographic structure of SLF (Kim et al., 2013; Park et al., 2013; Zhang et al., 2019). The comprehensive sampling in our phylogenetic analyses has successfully resolved this question. Six phylogeographic lineages were detected, which were consistent with the distribution of main Chinese topographical regions (i.e., East Plain, Loess Plateau, Sichuan Basin, Southeast Hills, Guizhou Plateau, and Yunnan Plateau). The Yangtze River is the most obvious geographic barrier for this species, shaping the divergence pattern between northern and southern Yangtze regions, which was also supported by previous studies (Kim et al., 2013; Zhang et al., 2019). Besides the Yangtze River, there were several other geographical barriers that caused the differentiation of SLF populations. The boundaries of the six topographical areas possibly impeded the gene flow of populations and facilitated the formation of different lineages. The distributions of these boundaries were accordant with the demarcation line of second and third steps of China's terrain and the drainage basin of Yangtze River. Based on the Mantel test, geographical distance played a pivotal role causing the genetic differentiation of SLF populations, indicating the historical range of expansion of this species. However, we also identified that specific Chinese populations such as Yinchuan, Ningxia (NXYC) which were located near the Loess Plateau regions but genetically, mostly contained individuals from the East Plain lineage. In this region, the SLF was first recorded in 2010, and its outbreak has occurred on the roadside *Ailanthus* trees since then (Zheng, 2018). It is likely that the SLF was introduced domestically through the timber trade and transportation between different provinces of China, which easily broke through the geographic barriers and facilitated the gene flow between these regions (Capinha et al., 2015; Zheng, 2018).

### 4.2 Origin, dispersal and demographic history

Combining the phylogenetic analyses and divergence time estimation, we inferred that SLF underwent a dispersal from southwestern to northeastern China during the late Pleistocene. SLF may have originated from Yunnan Plateau, not from northern China as initially thought (Liu, 1939). Southwestern China, especially Eastern Himalaya and Hengduan Mountains regions, has long been regarded as one of the most biodiverse regions in East Asia and the origin of different organisms including many insect species (Song et al., 2018; Wei et al., 2015). Out of Yunnan Plateau, SLF spread to the southern and northern Yangtze regions at 482.5 ka. The divergence of the southern Yangtze lineage into Guizhou Plateau and Southeast Hills lineages occurred at 191.4 ka, whereas the divergence of the northern Yangtze lineages including East Plain, Loess Plateau, and Sichuan Basin lineages occurred more recently, at 58.5–72.1 ka. Of the six regional populations, East Plain and Southeast Hills lineages experienced population expansion during the last 8 and 65 ka, respectively, while others have retained constant population sizes. The expansion of the Southeast Hills and East Plain lineages may have been associated with the spread across Yangtze River in the late Pleistocene and warming in the Holocene.

The population localities in the Guizhou Plateau and Yunnan Plateau regions were included in population genetic study for the first time (Kim et al., 2013; Zhang et al., 2019). The lack of these two pivotal lineages in previous studies resulted in an inaccurate understanding of origin and dispersal history of SLF (Liu, 1939; Zhang et al., 2019). Thus, comprehensive sampling should always be implemented as far as possible to reveal the phylogeographic structure and demographic history of a native species.

As the East Plain regions, including Northeast China Plain, North China Plain, and Middle‐Lower Yangtze Plain, harbors abundant SLF populations, it has long been recognized as the ancestral distribution area (Dara et al., 2015; Han et al., 2008; Liu, 1939). However, the East Plain lineage had the most recent divergence time among the six lineages. The warming event during Holocene possibly accounts for the quick expansion history, with the lack of geographic barrier and appropriate environmental conditions in this region. However, another crucial factor is the host plant, tree‐of‐heaven, the favorable and primary host for SLF both in China and the invasive countries, though more than 70 plants have been recorded as alternative hosts (Dara et al., 2015). Tree‐of‐heaven has been thought to be native to northeastern and central China and is commonly distributed in the Yellow River and Yangtze River basin areas as border trees and protection forests (Hu, 1979). The SLF is particularly attracted to tree‐of‐heaven and its wide distribution may have aided the quick dispersal of SLF populations in the East Plain region.

### 4.3 Global invasion history

Spotted lanternfly originating from southwestern China has now invaded three countries, including South Korea, Japan, and the United States successively. From our phylogenetic and network analyses, we found the populations of South Korea comprised two different genetic sources from East Plain (EP) and Loess Plateau lineages, whereas each of the United States and Japan had only one. Although the Korean Peninsula is bordering on northeast China in land, our network results did not support the dispersal of SLF across the land from northeast China, because the mutation step between northeast China and South Korea was large (Figure 4a; Northeast EP vs. South Korea haplotypes). Several shared haplotypes and fewer mutation steps between eastern China (Middle and South EP in Figure 4a) and South Korea implied the invasive routes across the sea, which was consistent with the earliest records and previous population genetic studies in South Korea (Han et al., 2008; Park et al., 2013). As the South Korea haplotypes were polyphyletic, multiple invasive events may have occurred in these regions through the continual sea transportation and trade, which commonly take place among East Asia countries (Havill et al., 2016; Kim et al., 2017).

Of all individuals in East Plain, five possessed haplotypes of Loess Plateau lineage, which suggest another route of SLF invasion from Loess Plateau to South Korea and Japan via East Plain. In addition, the only one South Korea haplotype in Loess Plateau lineage motivated us to track the invasion scenario between Japan and South Korea, because it had the sister relationship with the monophyletic Japan haplotypes. When SLF was first recorded in South Korea, researchers had predicted that it would be introduced into Japan soon, and the first Japan record was obtained three years thereafter. A single invasive event from a small population of South Korea with the haplotypes of Loess Plateau lineage may be a reasonable explanation for the origin of Japan population. However, considering the higher haplotype diversity in Japan (Table 1) and the connected relationship between haplotypes of East Plain and South Korea (Figure 4b) in our present study, we are more inclined to the alternative scenario of an invasion from eastern China to Japan, and subsequent dispersal to South Korea through a single introduction event.

The genetic source of population in the United States was inferred to be East Plain lineage. The lowest genetic diversity supported the single introduction hypothesis, and their closest relationships with South Korea haplotypes suggested the origin of the introduced population in the United States to be South Korea. In our study, South Korea was inferred to serve as a bridgehead in the global invasion history of SLF. The similar invasive pattern was also found in the soybean aphid (Kim et al., 2017). Even though large‐scale spatial sampling has been performed in China, there is a possibility that populations in United States were introduced directly from one or more undetected sources in eastern China, given the reduced mutations between them. Further sampling in this region and South Korea might provide a solution. Although no information has been available for the actual invasion process of SLF, the close geographical distance and frequent seaborne trade between Shandong and Jiangsu Provinces in eastern costal China, western coast of South Korea, and Japan may have facilitated the introduction of eggs, nymphs, or adults of SLF through wood. The environmental similarities and continuous trade between North America and East Asia may have facilitated invasions across the Pacific Ocean (Kim et al., 2017).

It is important to note that in the invasion history of SLF, the introduced population expanded quickly outward in South Korea (Han et al., 2008) and the United States (NYSIPM, 2020), even though a quarantine zone surrounding the site of first detection had been established. Tree‐of‐heaven was first introduced to the Philadelphia area of the United States in 1784 (Shah, 1997) and was widely distributed in South Korea and Japan before the invasion of SLF (Shimizu, 2003). The earlier invasion of tree‐of‐heaven in these countries possibly facilitated the quick invasion, colonization, and spread of SLF. The absence of natural enemies (Clifton et al., 2019) and suitable climatic conditions further contributed to the dispersal and damage of SLF in the invaded countries. Other regions such as many European countries have experienced the invasion of tree‐of‐heaven since long before (Hu, 1979) and are thus, possibly exposed to a high risk of outbreak. Therefore, monitoring and quarantining invasive SLF will be essential in these regions.

### 4.4 Implications for biological invasions

Biological invasion represents one of the natural rapid evolutionary processes in contemporary time scales. The genetic diversity of introduced populations might increase largely due to different invasion scenarios (Garnas et al., 2016). In our study, South Korea was inferred to serve as a bridgehead of global invasion, resulting in the introduction of SLF to the United States. Bridgehead effect has been considered as a pivotal factor to explain increasingly successful global invasions (Ascunce & Shoemaker, 2011; Javal et al., 2019). Nevertheless, little is known about how bridgehead effects facilitate secondary invasion. Bridgehead population may experience rapid evolution and possess higher adaptive abilities, which may lead to higher invasion capability. However, evidence supporting these hypotheses remains lacking (Bertelsmeier & Keller, 2018). The invading SLF population in the United States shows extremely low genetic diversity, but is currently undergoing rapid spread across eastern states, exhibiting exceptional adaptability to the new environment. This is a remarkable case of successful biological invasion with bridgehead effect and low genetic diversity, which can be established as a model to explore mechanisms behind the enhanced invasiveness in bridgehead populations. However, as discussed in the above sections, adaptation is not always necessary for outward establishment from the native ranges, because the introduced habitats generally have similar environmental conditions, especially host plants.

## 5 CONCLUSIONS AND FUTURE DIRECTIONS

Based on the detailed analysis of mitogenomic phylogeography, we explored the invasion history of SLF from China, to South Korea, Japan, and the United States. We revealed that the invasion of SLF into the United States may be a secondary invasion from a bridgehead population in South Korea. Our study supports the great potential of mass mitogenome sequences for resolving not only the phylogeographic pattern but also the invasive source and route of the pest species. Using high‐throughput sequencing and metagenomic approach, multiple invasive species could be analyzed together to reveal shared or different invasive patterns in defined regions. Mitogenome normally under strong purifying selection pressure offers little information for key genes behind invasion. Nevertheless, with whole genome sequence of SLF currently available (Kingan et al., 2019), a clear and accurate genetic structure derived from mitogenomic analysis may build a foundation for future studies on genomic mechanisms mediating rapid worldwide spread of this severe invasive pest.

## CONFLICTS OF INTEREST

The authors declare no conflict of interest.

## AUTHOR CONTRIBUTIONS

H.L. and W.C. designed the study. Z.D., Y.W., Z.C., L.C., T.I., S.K., and F.S. contributed to the sample collection. Z.D. performed the molecular experiments and phylogenomic analyses. All authors discussed the results, and Z.D., T.S., L.T., W.C., and H.L. wrote the paper.

## Supporting information

Supplementary Material

## Data Availability

All mitochondrial genome sequences used in this study have been deposited to GenBank (accession numbers: MT079333–MT079725).
