# Supplementary material for: Global phylogeography and invasion history of the spotted lanternfly revealed by mitochondrial phylogenomics
Source: Evol Appl. 2020 Dec 14;14(4):915–30. doi: 10.1111/eva.13170 (PMC8061274; doi:10.1111/eva.13170)
Supplement: Supplementary file 1 — Supplementary Material [file EVA-14-915-s001.docx]

**Fig. S1 Mantel tests between geographic distance and genetic differentiation of SLF.**

**
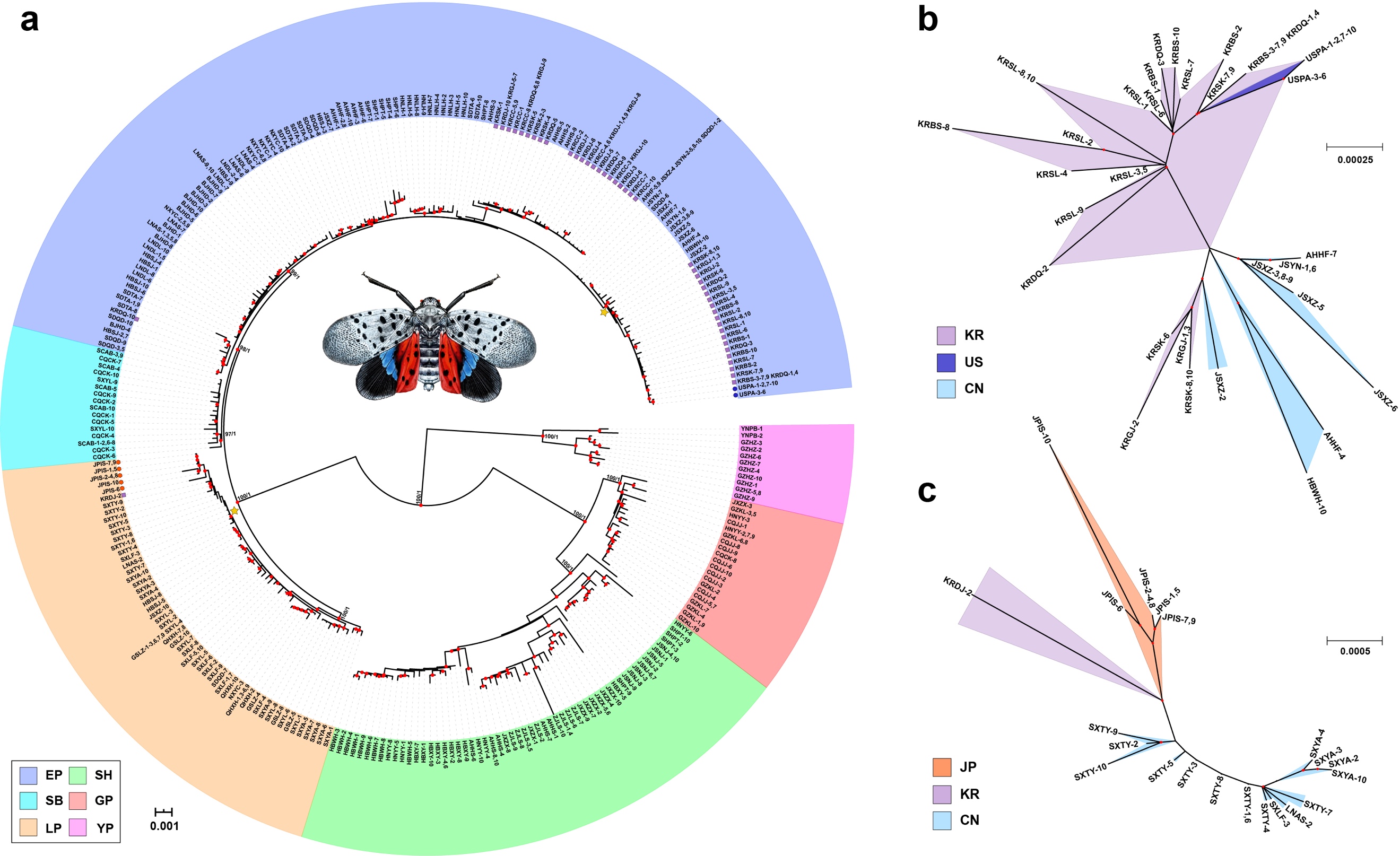
**

**Fig. S2 Phylogenetic relationships with branch length and two major clades with invasive haplotypes. a.** The phylogenic topology based on mitogenomic haplotypes, red circles represent the inside node with bootstrap values larger than 70. **b.** The clades with American and part South Korean haplotypes in the EP lineage. **c.** The clade with Japanese and single South Korean haplotypes in the LP lineage.

**Table S1** Pairwise *F_st_* values among 40 populations of SLF.

| Code | SDQD | SDTA | BJHD | SXTY | SXLF | HNLH | SXYL | SXYA | HBSJ | SHPT | GSLZ | QHXH | LNDL | LNAS | AHHF | AHHS | HNYY | HBWH | HBXY | GZHZ | GZKL | JXZX | ZJLS | JSXZ | JSYN | JSNJ | NXYC | CQJJ | CQCK | SCAB | YNPB | USPA | KRSL | KRSK | KRGJ | KRBS | KRDJ | KRCC | KRDQ | JPIS |
| --- | --- | --- | --- | --- | --- | --- | --- | --- | --- | --- | --- | --- | --- | --- | --- | --- | --- | --- | --- | --- | --- | --- | --- | --- | --- | --- | --- | --- | --- | --- | --- | --- | --- | --- | --- | --- | --- | --- | --- | --- |
| SDQD | 0.0000 |  |  |  |  |  |  |  |  |  |  |  |  |  |  |  |  |  |  |  |  |  |  |  |  |  |  |  |  |  |  |  |  |  |  |  |  |  |  |  |
| SDTA | 0.0368 | 0.0000 |  |  |  |  |  |  |  |  |  |  |  |  |  |  |  |  |  |  |  |  |  |  |  |  |  |  |  |  |  |  |  |  |  |  |  |  |  |  |
| BJHD | 0.2151 | 0.2496 | 0.0000 |  |  |  |  |  |  |  |  |  |  |  |  |  |  |  |  |  |  |  |  |  |  |  |  |  |  |  |  |  |  |  |  |  |  |  |  |  |
| SXTY | 0.6620 | 0.6990 | 0.7308 | 0.0000 |  |  |  |  |  |  |  |  |  |  |  |  |  |  |  |  |  |  |  |  |  |  |  |  |  |  |  |  |  |  |  |  |  |  |  |  |
| SXLF | 0.6190 | 0.6633 | 0.6922 | 0.1077 | 0.0000 |  |  |  |  |  |  |  |  |  |  |  |  |  |  |  |  |  |  |  |  |  |  |  |  |  |  |  |  |  |  |  |  |  |  |  |
| HNLH | 0.2168 | 0.2285 | 0.4446 | 0.7558 | 0.7248 | 0.0000 |  |  |  |  |  |  |  |  |  |  |  |  |  |  |  |  |  |  |  |  |  |  |  |  |  |  |  |  |  |  |  |  |  |  |
| SXYL | 0.4069 | 0.4668 | 0.4805 | 0.1725 | 0.1384 | 0.5151 | 0.0000 |  |  |  |  |  |  |  |  |  |  |  |  |  |  |  |  |  |  |  |  |  |  |  |  |  |  |  |  |  |  |  |  |  |
| SXYA | 0.5075 | 0.5601 | 0.5830 | 0.1790 | 0.1525 | 0.6086 | 0.0599 | 0.0000 |  |  |  |  |  |  |  |  |  |  |  |  |  |  |  |  |  |  |  |  |  |  |  |  |  |  |  |  |  |  |  |  |
| HBSJ | 0.0621 | 0.1514 | 0.0683 | 0.5734 | 0.5254 | 0.3322 | 0.3055 | 0.4179 | 0.0000 |  |  |  |  |  |  |  |  |  |  |  |  |  |  |  |  |  |  |  |  |  |  |  |  |  |  |  |  |  |  |  |
| SHPT | 0.3441 | 0.3463 | 0.3588 | 0.4067 | 0.4024 | 0.3452 | 0.3640 | 0.3808 | 0.3461 | 0.0000 |  |  |  |  |  |  |  |  |  |  |  |  |  |  |  |  |  |  |  |  |  |  |  |  |  |  |  |  |  |  |
| GSLZ | 0.6469 | 0.6819 | 0.7099 | 0.3039 | 0.2551 | 0.7324 | 0.0375 | 0.2239 | 0.5512 | 0.4075 | 0.0000 |  |  |  |  |  |  |  |  |  |  |  |  |  |  |  |  |  |  |  |  |  |  |  |  |  |  |  |  |  |
| QHXH | 0.7143 | 0.7402 | 0.7753 | 0.4558 | 0.3260 | 0.8136 | 0.2750 | 0.3614 | 0.6218 | 0.4204 | 0.4365 | 0.0000 |  |  |  |  |  |  |  |  |  |  |  |  |  |  |  |  |  |  |  |  |  |  |  |  |  |  |  |  |
| LNDL | 0.3245 | 0.3506 | 0.0778 | 0.7985 | 0.7575 | 0.5526 | 0.5254 | 0.6424 | 0.1306 | 0.3677 | 0.7708 | 0.8463 | 0.0000 |  |  |  |  |  |  |  |  |  |  |  |  |  |  |  |  |  |  |  |  |  |  |  |  |  |  |  |
| LNAS | 0.2372 | 0.2783 | 0.0645 | 0.6785 | 0.6379 | 0.4370 | 0.4237 | 0.5266 | 0.0766 | 0.3553 | 0.6619 | 0.7293 | 0.1215 | 0.0000 |  |  |  |  |  |  |  |  |  |  |  |  |  |  |  |  |  |  |  |  |  |  |  |  |  |  |
| AHHF | 0.2469 | 0.2431 | 0.4773 | 0.8002 | 0.7640 | 0.2456 | 0.5540 | 0.6572 | 0.3525 | 0.3509 | 0.7744 | 0.8469 | 0.6020 | 0.4743 | 0.0000 |  |  |  |  |  |  |  |  |  |  |  |  |  |  |  |  |  |  |  |  |  |  |  |  |  |
| AHHS | 0.5171 | 0.5184 | 0.5250 | 0.5474 | 0.5432 | 0.5206 | 0.5139 | 0.5280 | 0.5148 | 0.0031 | 0.5452 | 0.5569 | 0.5336 | 0.5213 | 0.5304 | 0.0000 |  |  |  |  |  |  |  |  |  |  |  |  |  |  |  |  |  |  |  |  |  |  |  |  |
| HNYY | 0.8663 | 0.8652 | 0.8693 | 0.8735 | 0.8703 | 0.8719 | 0.8500 | 0.8612 | 0.8620 | 0.4405 | 0.8703 | 0.8786 | 0.8760 | 0.8668 | 0.8758 | 0.2675 | 0.0000 |  |  |  |  |  |  |  |  |  |  |  |  |  |  |  |  |  |  |  |  |  |  |  |
| HBWH | 0.8625 | 0.8612 | 0.8659 | 0.8714 | 0.8678 | 0.8684 | 0.8460 | 0.8580 | 0.8580 | 0.4126 | 0.8679 | 0.8768 | 0.8733 | 0.8632 | 0.8725 | 0.2407 | 0.1709 | 0.0000 |  |  |  |  |  |  |  |  |  |  |  |  |  |  |  |  |  |  |  |  |  |  |
| HBXY | 0.9609 | 0.9592 | 0.9639 | 0.9670 | 0.9637 | 0.9664 | 0.9434 | 0.9547 | 0.9565 | 0.5632 | 0.9633 | 0.9713 | 0.9709 | 0.9615 | 0.9698 | 0.3966 | 0.2935 | 0.0082 | 0.0000 |  |  |  |  |  |  |  |  |  |  |  |  |  |  |  |  |  |  |  |  |  |
| GZHZ | 0.9457 | 0.9442 | 0.9482 | 0.9505 | 0.9479 | 0.9502 | 0.9310 | 0.9407 | 0.9421 | 0.7502 | 0.9476 | 0.9541 | 0.9537 | 0.9461 | 0.9531 | 0.7464 | 0.8760 | 0.8830 | 0.9465 | 0.0000 |  |  |  |  |  |  |  |  |  |  |  |  |  |  |  |  |  |  |  |  |
| GZKL | 0.9583 | 0.9566 | 0.9614 | 0.9648 | 0.9613 | 0.9641 | 0.9401 | 0.9519 | 0.9538 | 0.5764 | 0.9609 | 0.9693 | 0.9688 | 0.9590 | 0.9677 | 0.4621 | 0.3717 | 0.6497 | 0.8530 | 0.9430 | 0.0000 |  |  |  |  |  |  |  |  |  |  |  |  |  |  |  |  |  |  |  |
| JXZX | 0.9139 | 0.9124 | 0.9169 | 0.9208 | 0.9174 | 0.9194 | 0.8969 | 0.9083 | 0.9095 | 0.4817 | 0.9172 | 0.9253 | 0.9239 | 0.9144 | 0.9232 | 0.2801 | 0.1366 | 0.2269 | 0.3683 | 0.9118 | 0.6690 | 0.0000 |  |  |  |  |  |  |  |  |  |  |  |  |  |  |  |  |  |  |
| ZJLS | 0.9324 | 0.9308 | 0.9355 | 0.9392 | 0.9357 | 0.9381 | 0.9145 | 0.9263 | 0.9278 | 0.4803 | 0.9354 | 0.9437 | 0.9428 | 0.9330 | 0.9419 | 0.2732 | 0.2979 | 0.3659 | 0.5705 | 0.9251 | 0.7517 | 0.2551 | 0.0000 |  |  |  |  |  |  |  |  |  |  |  |  |  |  |  |  |  |
| JSXZ | 0.0722 | 0.1098 | 0.3291 | 0.6986 | 0.6577 | 0.1199 | 0.4413 | 0.5476 | 0.1723 | 0.3438 | 0.6749 | 0.7477 | 0.4279 | 0.3251 | 0.1457 | 0.5199 | 0.8688 | 0.8651 | 0.9632 | 0.9476 | 0.9607 | 0.9163 | 0.9349 | 0.0000 |  |  |  |  |  |  |  |  |  |  |  |  |  |  |  |  |
| JSYN | 0.2107 | 0.2014 | 0.5351 | 0.8701 | 0.8280 | 0.2698 | 0.5918 | 0.7113 | 0.3606 | 0.3610 | 0.8358 | 0.9197 | 0.7325 | 0.5269 | 0.3281 | 0.5358 | 0.8822 | 0.8797 | 0.9771 | 0.9590 | 0.9754 | 0.9302 | 0.9493 | 0.0163 | 0.0000 |  |  |  |  |  |  |  |  |  |  |  |  |  |  |  |
| JSNJ | 0.9624 | 0.9607 | 0.9655 | 0.9688 | 0.9653 | 0.9681 | 0.9444 | 0.9561 | 0.9579 | 0.5245 | 0.9648 | 0.9730 | 0.9727 | 0.9631 | 0.9717 | 0.3258 | 0.3624 | 0.4343 | 0.6983 | 0.9468 | 0.8398 | 0.3531 | 0.1064 | 0.9648 | 0.9792 | 0.0000 |  |  |  |  |  |  |  |  |  |  |  |  |  |  |
| NXYC | 0.2709 | 0.3116 | 0.1383 | 0.6907 | 0.6491 | 0.4684 | 0.4420 | 0.5424 | 0.1168 | 0.3596 | 0.6724 | 0.7293 | 0.1811 | 0.1214 | 0.4976 | 0.5243 | 0.8679 | 0.8644 | 0.9619 | 0.9467 | 0.9595 | 0.9151 | 0.9334 | 0.3540 | 0.5537 | 0.9634 | 0.0000 |  |  |  |  |  |  |  |  |  |  |  |  |  |
| CQJJ | 0.9565 | 0.9548 | 0.9596 | 0.9630 | 0.9595 | 0.9622 | 0.9383 | 0.9502 | 0.9520 | 0.5750 | 0.9591 | 0.9675 | 0.9670 | 0.9572 | 0.9659 | 0.4615 | 0.3537 | 0.6437 | 0.8458 | 0.9417 | 0.0340 | 0.6610 | 0.7479 | 0.9589 | 0.9735 | 0.8347 | 0.9577 | 0.0000 |  |  |  |  |  |  |  |  |  |  |  |  |
| CQCK | 0.1581 | 0.1807 | 0.1761 | 0.3166 | 0.3012 | 0.2182 | 0.1770 | 0.2428 | 0.1318 | 0.2044 | 0.3224 | 0.3464 | 0.1796 | 0.1660 | 0.2314 | 0.3685 | 0.7501 | 0.7435 | 0.8556 | 0.8662 | 0.8437 | 0.8025 | 0.8200 | 0.1840 | 0.2271 | 0.8535 | 0.1831 | 0.8419 | 0.0000 |  |  |  |  |  |  |  |  |  |  |  |
| SCAB | 0.5496 | 0.5660 | 0.5966 | 0.7532 | 0.7183 | 0.6614 | 0.4747 | 0.5982 | 0.4822 | 0.3811 | 0.7306 | 0.8017 | 0.6640 | 0.5714 | 0.7053 | 0.5333 | 0.8708 | 0.8685 | 0.9656 | 0.9487 | 0.9632 | 0.9187 | 0.9371 | 0.5993 | 0.7854 | 0.9671 | 0.5770 | 0.9614 | 0.0412 | 0.0000 |  |  |  |  |  |  |  |  |  |  |
| YNPB | 0.9732 | 0.9706 | 0.9775 | 0.9814 | 0.9766 | 0.9810 | 0.9479 | 0.9641 | 0.9671 | 0.6579 | 0.9759 | 0.9871 | 0.9874 | 0.9742 | 0.9857 | 0.6510 | 0.8500 | 0.8616 | 0.9676 | 0.7765 | 0.9648 | 0.9095 | 0.9330 | 0.9764 | 0.9962 | 0.9698 | 0.9746 | 0.9625 | 0.8425 | 0.9796 | 0.0000 |  |  |  |  |  |  |  |  |  |
| USPA | 0.5405 | 0.5296 | 0.7011 | 0.8781 | 0.8443 | 0.5132 | 0.6289 | 0.7327 | 0.5714 | 0.3708 | 0.8451 | 0.9306 | 0.8409 | 0.6741 | 0.6998 | 0.5401 | 0.8829 | 0.8805 | 0.9772 | 0.9592 | 0.9755 | 0.9306 | 0.9496 | 0.5170 | 1.0000 | 0.9793 | 0.6947 | 0.9736 | 0.2898 | 0.8260 | 0.9962 | 0.0000 |  |  |  |  |  |  |  |  |
| KRSL | 0.1541 | 0.1686 | 0.4350 | 0.7942 | 0.7568 | 0.1197 | 0.5402 | 0.6458 | 0.3042 | 0.3507 | 0.7680 | 0.8427 | 0.5652 | 0.4359 | 0.1717 | 0.5269 | 0.8753 | 0.8723 | 0.9699 | 0.9530 | 0.9677 | 0.9230 | 0.9417 | 0.0278 | 0.0741 | 0.9716 | 0.4639 | 0.9658 | 0.2148 | 0.6920 | 0.9858 | 0.6612 | 0.0000 |  |  |  |  |  |  |  |
| KRSK | 0.3191 | 0.3278 | 0.5587 | 0.8243 | 0.7900 | 0.0903 | 0.5612 | 0.6665 | 0.4219 | 0.3514 | 0.7934 | 0.8850 | 0.7026 | 0.5385 | 0.4064 | 0.5283 | 0.8784 | 0.8756 | 0.9735 | 0.9560 | 0.9715 | 0.9264 | 0.9454 | 0.2273 | 0.6296 | 0.9755 | 0.5702 | 0.9697 | 0.2357 | 0.7499 | 0.9912 | 0.7436 | 0.2912 | 0.0000 |  |  |  |  |  |  |
| KRGJ | 0.2360 | 0.2295 | 0.5119 | 0.8214 | 0.7847 | 0.0432 | 0.5524 | 0.6626 | 0.3683 | 0.3510 | 0.7908 | 0.8792 | 0.6616 | 0.4953 | 0.2973 | 0.5286 | 0.8783 | 0.8754 | 0.9733 | 0.9557 | 0.9713 | 0.9262 | 0.9452 | 0.0851 | 0.4074 | 0.9753 | 0.5310 | 0.9694 | 0.2221 | 0.7359 | 0.9908 | 0.7461 | 0.1322 | 0.1389 | 0.0000 |  |  |  |  |  |
| KRBS | 0.2854 | 0.2898 | 0.5029 | 0.7908 | 0.7579 | 0.1640 | 0.5462 | 0.6453 | 0.3832 | 0.3514 | 0.7650 | 0.8455 | 0.6223 | 0.4920 | 0.3489 | 0.5276 | 0.8751 | 0.8720 | 0.9695 | 0.9528 | 0.9673 | 0.9227 | 0.9414 | 0.1191 | 0.4359 | 0.9713 | 0.5201 | 0.9655 | 0.2346 | 0.7055 | 0.9853 | 0.6508 | 0.2485 | 0.2889 | 0.1559 | 0.0000 |  |  |  |  |
| KRDJ | 0.0656 | 0.1013 | 0.3261 | 0.6931 | 0.6530 | 0.0741 | 0.4351 | 0.5408 | 0.1736 | 0.3424 | 0.6695 | 0.7452 | 0.4245 | 0.3207 | 0.1337 | 0.5184 | 0.8685 | 0.8648 | 0.9630 | 0.9474 | 0.9605 | 0.9161 | 0.9346 | -0.0499 | 0.0081 | 0.9646 | 0.3513 | 0.9587 | 0.1809 | 0.5954 | 0.9762 | 0.4945 | 0.0033 | 0.1842 | 0.0637 | 0.1643 | 0.0000 |  |  |  |
| KRCC | 0.1788 | 0.1769 | 0.4415 | 0.7876 | 0.7516 | 0.0933 | 0.5341 | 0.6381 | 0.3135 | 0.3499 | 0.7616 | 0.8395 | 0.5685 | 0.4389 | 0.2125 | 0.5266 | 0.8750 | 0.8718 | 0.9695 | 0.9527 | 0.9673 | 0.9226 | 0.9414 | 0.0118 | 0.1438 | 0.9713 | 0.4689 | 0.9655 | 0.2158 | 0.6884 | 0.9853 | 0.6268 | 0.0643 | 0.2140 | 0.0240 | 0.1036 | 0.0252 | 0.0000 |  |  |
| KRDQ | 0.0554 | 0.0974 | 0.3079 | 0.7165 | 0.6834 | 0.1333 | 0.4744 | 0.5701 | 0.2003 | 0.3420 | 0.6966 | 0.7709 | 0.4180 | 0.3203 | 0.2346 | 0.5181 | 0.8682 | 0.8646 | 0.9628 | 0.9473 | 0.9603 | 0.9158 | 0.9344 | 0.0499 | 0.2221 | 0.9644 | 0.3611 | 0.9584 | 0.1859 | 0.5991 | 0.9759 | 0.5050 | 0.1363 | 0.2119 | 0.1058 | 0.0566 | 0.0816 | 0.0675 | 0.0000 |  |
| JPIS | 0.7050 | 0.7324 | 0.7677 | 0.4004 | 0.3373 | 0.7889 | 0.1771 | 0.3300 | 0.6056 | 0.4170 | 0.1982 | 0.5724 | 0.8356 | 0.7215 | 0.8326 | 0.5540 | 0.8768 | 0.8749 | 0.9698 | 0.9530 | 0.9677 | 0.9238 | 0.9423 | 0.7312 | 0.9046 | 0.9716 | 0.7321 | 0.9659 | 0.3424 | 0.7888 | 0.9852 | 0.9104 | 0.8279 | 0.8602 | 0.8573 | 0.8243 | 0.7262 | 0.8217 | 0.7517 | 0.0000 |

Underlines mean not significant statistically (P > 0.05).

**Table S2** Pairwise *p*-distance among 40 populations of SLF.

| Code | SDQD | SDTA | BJHD | SXTY | SXLF | HNLH | SXYL | SXYA | HBSJ | SHPT | GSLZ | QHXH | LNDL | LNAS | AHHF | AHHS | HNYY | HBWH | HBXY | GZHZ | GZKL | JXZX | ZJLS | JSXZ | JSYN | JSNJ | NXYC | CQJJ | CQCK | SCAB | YNPB | USPA | KRSL | KRSK | KRGJ | KRBS | KRDJ | KRCC | KRDQ | JPIS |
| --- | --- | --- | --- | --- | --- | --- | --- | --- | --- | --- | --- | --- | --- | --- | --- | --- | --- | --- | --- | --- | --- | --- | --- | --- | --- | --- | --- | --- | --- | --- | --- | --- | --- | --- | --- | --- | --- | --- | --- | --- |
| SDQD | 0.0000 |  |  |  |  |  |  |  |  |  |  |  |  |  |  |  |  |  |  |  |  |  |  |  |  |  |  |  |  |  |  |  |  |  |  |  |  |  |  |  |
| SDTA | 0.0005 | 0.0000 |  |  |  |  |  |  |  |  |  |  |  |  |  |  |  |  |  |  |  |  |  |  |  |  |  |  |  |  |  |  |  |  |  |  |  |  |  |  |
| BJHD | 0.0005 | 0.0006 | 0.0000 |  |  |  |  |  |  |  |  |  |  |  |  |  |  |  |  |  |  |  |  |  |  |  |  |  |  |  |  |  |  |  |  |  |  |  |  |  |
| SXTY | 0.0011 | 0.0013 | 0.0012 | 0.0000 |  |  |  |  |  |  |  |  |  |  |  |  |  |  |  |  |  |  |  |  |  |  |  |  |  |  |  |  |  |  |  |  |  |  |  |  |
| SXLF | 0.0011 | 0.0013 | 0.0012 | 0.0004 | 0.0000 |  |  |  |  |  |  |  |  |  |  |  |  |  |  |  |  |  |  |  |  |  |  |  |  |  |  |  |  |  |  |  |  |  |  |  |
| HNLH | 0.0005 | 0.0005 | 0.0006 | 0.0012 | 0.0012 | 0.0000 |  |  |  |  |  |  |  |  |  |  |  |  |  |  |  |  |  |  |  |  |  |  |  |  |  |  |  |  |  |  |  |  |  |  |
| SXYL | 0.0011 | 0.0013 | 0.0012 | 0.0007 | 0.0007 | 0.0012 | 0.0000 |  |  |  |  |  |  |  |  |  |  |  |  |  |  |  |  |  |  |  |  |  |  |  |  |  |  |  |  |  |  |  |  |  |
| SXYA | 0.0011 | 0.0012 | 0.0012 | 0.0005 | 0.0006 | 0.0011 | 0.0008 | 0.0000 |  |  |  |  |  |  |  |  |  |  |  |  |  |  |  |  |  |  |  |  |  |  |  |  |  |  |  |  |  |  |  |  |
| HBSJ | 0.0005 | 0.0006 | 0.0005 | 0.0010 | 0.0010 | 0.0006 | 0.0011 | 0.0010 | 0.0000 |  |  |  |  |  |  |  |  |  |  |  |  |  |  |  |  |  |  |  |  |  |  |  |  |  |  |  |  |  |  |  |
| SHPT | 0.0056 | 0.0056 | 0.0056 | 0.0060 | 0.0061 | 0.0055 | 0.0061 | 0.0061 | 0.0057 | 0.0000 |  |  |  |  |  |  |  |  |  |  |  |  |  |  |  |  |  |  |  |  |  |  |  |  |  |  |  |  |  |  |
| GSLZ | 0.0012 | 0.0014 | 0.0013 | 0.0005 | 0.0005 | 0.0013 | 0.0007 | 0.0006 | 0.0010 | 0.0061 | 0.0000 |  |  |  |  |  |  |  |  |  |  |  |  |  |  |  |  |  |  |  |  |  |  |  |  |  |  |  |  |  |
| QHXH | 0.0011 | 0.0013 | 0.0012 | 0.0004 | 0.0004 | 0.0012 | 0.0008 | 0.0006 | 0.0010 | 0.0061 | 0.0005 | 0.0000 |  |  |  |  |  |  |  |  |  |  |  |  |  |  |  |  |  |  |  |  |  |  |  |  |  |  |  |  |
| LNDL | 0.0004 | 0.0005 | 0.0003 | 0.0011 | 0.0011 | 0.0005 | 0.0011 | 0.0011 | 0.0004 | 0.0056 | 0.0012 | 0.0011 | 0.0000 |  |  |  |  |  |  |  |  |  |  |  |  |  |  |  |  |  |  |  |  |  |  |  |  |  |  |  |
| LNAS | 0.0006 | 0.0006 | 0.0004 | 0.0011 | 0.0011 | 0.0006 | 0.0012 | 0.0011 | 0.0005 | 0.0057 | 0.0012 | 0.0011 | 0.0003 | 0.0000 |  |  |  |  |  |  |  |  |  |  |  |  |  |  |  |  |  |  |  |  |  |  |  |  |  |  |
| AHHF | 0.0004 | 0.0004 | 0.0005 | 0.0012 | 0.0012 | 0.0003 | 0.0012 | 0.0012 | 0.0006 | 0.0055 | 0.0013 | 0.0012 | 0.0005 | 0.0006 | 0.0000 |  |  |  |  |  |  |  |  |  |  |  |  |  |  |  |  |  |  |  |  |  |  |  |  |  |
| AHHS | 0.0078 | 0.0079 | 0.0079 | 0.0082 | 0.0082 | 0.0077 | 0.0083 | 0.0082 | 0.0079 | 0.0070 | 0.0083 | 0.0082 | 0.0078 | 0.0079 | 0.0078 | 0.0000 |  |  |  |  |  |  |  |  |  |  |  |  |  |  |  |  |  |  |  |  |  |  |  |  |
| HNYY | 0.0129 | 0.0129 | 0.0129 | 0.0130 | 0.0130 | 0.0129 | 0.0131 | 0.0130 | 0.0129 | 0.0089 | 0.0131 | 0.0131 | 0.0128 | 0.0128 | 0.0129 | 0.0069 | 0.0000 |  |  |  |  |  |  |  |  |  |  |  |  |  |  |  |  |  |  |  |  |  |  |  |
| HBWH | 0.0117 | 0.0118 | 0.0117 | 0.0120 | 0.0120 | 0.0117 | 0.0120 | 0.0120 | 0.0118 | 0.0083 | 0.0120 | 0.0121 | 0.0117 | 0.0117 | 0.0118 | 0.0065 | 0.0035 | 0.0000 |  |  |  |  |  |  |  |  |  |  |  |  |  |  |  |  |  |  |  |  |  |  |
| HBXY | 0.0130 | 0.0131 | 0.0130 | 0.0131 | 0.0132 | 0.0130 | 0.0132 | 0.0132 | 0.0130 | 0.0086 | 0.0132 | 0.0132 | 0.0130 | 0.0130 | 0.0130 | 0.0064 | 0.0026 | 0.0017 | 0.0000 |  |  |  |  |  |  |  |  |  |  |  |  |  |  |  |  |  |  |  |  |  |
| GZHZ | 0.0158 | 0.0158 | 0.0158 | 0.0159 | 0.0159 | 0.0158 | 0.0159 | 0.0160 | 0.0158 | 0.0163 | 0.0160 | 0.0159 | 0.0157 | 0.0158 | 0.0159 | 0.0165 | 0.0173 | 0.0174 | 0.0176 | 0.0000 |  |  |  |  |  |  |  |  |  |  |  |  |  |  |  |  |  |  |  |  |
| GZKL | 0.0124 | 0.0125 | 0.0124 | 0.0125 | 0.0126 | 0.0124 | 0.0126 | 0.0126 | 0.0124 | 0.0089 | 0.0126 | 0.0126 | 0.0123 | 0.0124 | 0.0124 | 0.0072 | 0.0029 | 0.0049 | 0.0041 | 0.0166 | 0.0000 |  |  |  |  |  |  |  |  |  |  |  |  |  |  |  |  |  |  |  |
| JXZX | 0.0127 | 0.0128 | 0.0127 | 0.0129 | 0.0129 | 0.0127 | 0.0129 | 0.0129 | 0.0127 | 0.0084 | 0.0129 | 0.0129 | 0.0126 | 0.0127 | 0.0128 | 0.0062 | 0.0028 | 0.0030 | 0.0019 | 0.0173 | 0.0036 | 0.0000 |  |  |  |  |  |  |  |  |  |  |  |  |  |  |  |  |  |  |
| ZJLS | 0.0124 | 0.0125 | 0.0124 | 0.0125 | 0.0125 | 0.0124 | 0.0126 | 0.0125 | 0.0124 | 0.0078 | 0.0126 | 0.0126 | 0.0123 | 0.0123 | 0.0124 | 0.0058 | 0.0031 | 0.0032 | 0.0022 | 0.0169 | 0.0038 | 0.0020 | 0.0000 |  |  |  |  |  |  |  |  |  |  |  |  |  |  |  |  |  |
| JSXZ | 0.0004 | 0.0005 | 0.0005 | 0.0011 | 0.0011 | 0.0004 | 0.0012 | 0.0011 | 0.0006 | 0.0055 | 0.0012 | 0.0011 | 0.0005 | 0.0006 | 0.0003 | 0.0078 | 0.0129 | 0.0118 | 0.0130 | 0.0159 | 0.0124 | 0.0127 | 0.0124 | 0.0000 |  |  |  |  |  |  |  |  |  |  |  |  |  |  |  |  |
| JSYN | 0.0003 | 0.0003 | 0.0004 | 0.0011 | 0.0011 | 0.0002 | 0.0011 | 0.0011 | 0.0004 | 0.0054 | 0.0012 | 0.0011 | 0.0003 | 0.0004 | 0.0001 | 0.0077 | 0.0128 | 0.0116 | 0.0129 | 0.0158 | 0.0123 | 0.0126 | 0.0123 | 0.0002 | 0.0000 |  |  |  |  |  |  |  |  |  |  |  |  |  |  |  |
| JSNJ | 0.0126 | 0.0127 | 0.0126 | 0.0127 | 0.0127 | 0.0126 | 0.0128 | 0.0128 | 0.0126 | 0.0078 | 0.0128 | 0.0128 | 0.0125 | 0.0126 | 0.0126 | 0.0057 | 0.0028 | 0.0030 | 0.0019 | 0.0170 | 0.0036 | 0.0018 | 0.0010 | 0.0126 | 0.0125 | 0.0000 |  |  |  |  |  |  |  |  |  |  |  |  |  |  |
| NXYC | 0.0006 | 0.0006 | 0.0004 | 0.0011 | 0.0011 | 0.0007 | 0.0012 | 0.0011 | 0.0005 | 0.0057 | 0.0012 | 0.0011 | 0.0003 | 0.0005 | 0.0006 | 0.0079 | 0.0129 | 0.0118 | 0.0130 | 0.0159 | 0.0124 | 0.0127 | 0.0124 | 0.0006 | 0.0005 | 0.0126 | 0.0000 |  |  |  |  |  |  |  |  |  |  |  |  |  |
| CQJJ | 0.0124 | 0.0125 | 0.0124 | 0.0126 | 0.0126 | 0.0124 | 0.0126 | 0.0126 | 0.0124 | 0.0089 | 0.0127 | 0.0127 | 0.0124 | 0.0124 | 0.0125 | 0.0072 | 0.0029 | 0.0049 | 0.0041 | 0.0167 | 0.0007 | 0.0036 | 0.0038 | 0.0124 | 0.0123 | 0.0036 | 0.0125 | 0.0000 |  |  |  |  |  |  |  |  |  |  |  |  |
| CQCK | 0.0020 | 0.0021 | 0.0020 | 0.0023 | 0.0024 | 0.0021 | 0.0023 | 0.0023 | 0.0020 | 0.0062 | 0.0024 | 0.0024 | 0.0019 | 0.0020 | 0.0020 | 0.0080 | 0.0119 | 0.0112 | 0.0122 | 0.0157 | 0.0113 | 0.0119 | 0.0116 | 0.0020 | 0.0019 | 0.0117 | 0.0020 | 0.0113 | 0.0000 |  |  |  |  |  |  |  |  |  |  |  |
| SCAB | 0.0008 | 0.0009 | 0.0008 | 0.0012 | 0.0012 | 0.0009 | 0.0012 | 0.0011 | 0.0008 | 0.0058 | 0.0013 | 0.0012 | 0.0007 | 0.0008 | 0.0009 | 0.0080 | 0.0128 | 0.0118 | 0.0130 | 0.0156 | 0.0124 | 0.0127 | 0.0123 | 0.0009 | 0.0007 | 0.0126 | 0.0008 | 0.0125 | 0.0017 | 0.0000 |  |  |  |  |  |  |  |  |  |  |
| YNPB | 0.0166 | 0.0166 | 0.0166 | 0.0166 | 0.0167 | 0.0166 | 0.0167 | 0.0167 | 0.0166 | 0.0171 | 0.0168 | 0.0167 | 0.0166 | 0.0166 | 0.0167 | 0.0173 | 0.0179 | 0.0182 | 0.0183 | 0.0054 | 0.0172 | 0.0179 | 0.0175 | 0.0167 | 0.0166 | 0.0175 | 0.0167 | 0.0173 | 0.0166 | 0.0165 | 0.0000 |  |  |  |  |  |  |  |  |  |
| USPA | 0.0005 | 0.0005 | 0.0006 | 0.0011 | 0.0012 | 0.0003 | 0.0012 | 0.0011 | 0.0006 | 0.0055 | 0.0012 | 0.0012 | 0.0005 | 0.0006 | 0.0003 | 0.0077 | 0.0129 | 0.0117 | 0.0130 | 0.0158 | 0.0124 | 0.0127 | 0.0124 | 0.0004 | 0.0002 | 0.0126 | 0.0007 | 0.0124 | 0.0021 | 0.0009 | 0.0166 | 0.0000 |  |  |  |  |  |  |  |  |
| KRSL | 0.0004 | 0.0004 | 0.0005 | 0.0012 | 0.0012 | 0.0003 | 0.0012 | 0.0011 | 0.0005 | 0.0055 | 0.0012 | 0.0012 | 0.0004 | 0.0005 | 0.0002 | 0.0077 | 0.0128 | 0.0117 | 0.0130 | 0.0158 | 0.0124 | 0.0127 | 0.0123 | 0.0003 | 0.0001 | 0.0126 | 0.0006 | 0.0124 | 0.0020 | 0.0008 | 0.0166 | 0.0003 | 0.0000 |  |  |  |  |  |  |  |
| KRSK | 0.0004 | 0.0004 | 0.0005 | 0.0011 | 0.0011 | 0.0002 | 0.0012 | 0.0011 | 0.0006 | 0.0054 | 0.0012 | 0.0012 | 0.0004 | 0.0005 | 0.0002 | 0.0076 | 0.0128 | 0.0116 | 0.0129 | 0.0157 | 0.0123 | 0.0126 | 0.0123 | 0.0003 | 0.0001 | 0.0125 | 0.0006 | 0.0123 | 0.0020 | 0.0008 | 0.0165 | 0.0002 | 0.0002 | 0.0000 |  |  |  |  |  |  |
| KRGJ | 0.0003 | 0.0004 | 0.0005 | 0.0011 | 0.0011 | 0.0002 | 0.0011 | 0.0010 | 0.0005 | 0.0054 | 0.0012 | 0.0011 | 0.0004 | 0.0005 | 0.0002 | 0.0077 | 0.0128 | 0.0116 | 0.0129 | 0.0157 | 0.0123 | 0.0126 | 0.0123 | 0.0003 | 0.0001 | 0.0125 | 0.0005 | 0.0123 | 0.0019 | 0.0008 | 0.0165 | 0.0002 | 0.0002 | 0.0001 | 0.0000 |  |  |  |  |  |
| KRBS | 0.0004 | 0.0005 | 0.0006 | 0.0012 | 0.0012 | 0.0003 | 0.0012 | 0.0011 | 0.0006 | 0.0055 | 0.0013 | 0.0012 | 0.0005 | 0.0006 | 0.0003 | 0.0078 | 0.0129 | 0.0117 | 0.0130 | 0.0158 | 0.0124 | 0.0127 | 0.0124 | 0.0003 | 0.0002 | 0.0126 | 0.0006 | 0.0124 | 0.0020 | 0.0009 | 0.0166 | 0.0003 | 0.0003 | 0.0002 | 0.0002 | 0.0000 |  |  |  |  |
| KRDJ | 0.0004 | 0.0005 | 0.0005 | 0.0011 | 0.0011 | 0.0004 | 0.0011 | 0.0011 | 0.0006 | 0.0055 | 0.0012 | 0.0011 | 0.0005 | 0.0006 | 0.0003 | 0.0078 | 0.0129 | 0.0117 | 0.0130 | 0.0158 | 0.0124 | 0.0127 | 0.0124 | 0.0004 | 0.0002 | 0.0126 | 0.0006 | 0.0124 | 0.0020 | 0.0008 | 0.0166 | 0.0004 | 0.0003 | 0.0003 | 0.0003 | 0.0003 | 0.0000 |  |  |  |
| KRCC | 0.0004 | 0.0004 | 0.0005 | 0.0011 | 0.0012 | 0.0003 | 0.0012 | 0.0011 | 0.0005 | 0.0055 | 0.0012 | 0.0012 | 0.0004 | 0.0005 | 0.0003 | 0.0077 | 0.0128 | 0.0117 | 0.0130 | 0.0158 | 0.0124 | 0.0127 | 0.0124 | 0.0003 | 0.0001 | 0.0126 | 0.0006 | 0.0124 | 0.0020 | 0.0008 | 0.0166 | 0.0003 | 0.0002 | 0.0002 | 0.0002 | 0.0002 | 0.0003 | 0.0000 |  |  |
| KRDQ | 0.0004 | 0.0005 | 0.0005 | 0.0012 | 0.0012 | 0.0004 | 0.0012 | 0.0012 | 0.0006 | 0.0055 | 0.0013 | 0.0012 | 0.0005 | 0.0006 | 0.0004 | 0.0078 | 0.0129 | 0.0117 | 0.0130 | 0.0158 | 0.0124 | 0.0127 | 0.0124 | 0.0004 | 0.0002 | 0.0126 | 0.0006 | 0.0124 | 0.0020 | 0.0009 | 0.0166 | 0.0004 | 0.0003 | 0.0003 | 0.0003 | 0.0003 | 0.0004 | 0.0003 | 0.0000 |  |
| JPIS | 0.0011 | 0.0013 | 0.0012 | 0.0004 | 0.0004 | 0.0012 | 0.0007 | 0.0006 | 0.0010 | 0.0061 | 0.0004 | 0.0004 | 0.0011 | 0.0011 | 0.0012 | 0.0082 | 0.0131 | 0.0120 | 0.0132 | 0.0159 | 0.0126 | 0.0129 | 0.0126 | 0.0011 | 0.0011 | 0.0128 | 0.0011 | 0.0126 | 0.0024 | 0.0012 | 0.0167 | 0.0012 | 0.0012 | 0.0011 | 0.0011 | 0.0012 | 0.0011 | 0.0012 | 0.0012 | 0.0000 |

**Table S3** Haplotype frequency of the EP lineage.

| Haplotype | AHHF | AHHS | BJHD | HBSJ | HBWH | HNLH | JSXZ | JSYN | KRBS | KRCC | KRDJ | KRDQ | KRGJ | KRSK | KRSL | LNAS | LNDL | NXYC | SDQD | SDTA | SHPT | USPA |
| --- | --- | --- | --- | --- | --- | --- | --- | --- | --- | --- | --- | --- | --- | --- | --- | --- | --- | --- | --- | --- | --- | --- |
| Hap1 | 1 | 0 | 0 | 0 | 0 | 0 | 0 | 0 | 0 | 0 | 0 | 0 | 0 | 0 | 0 | 0 | 0 | 0 | 0 | 0 | 0 | 0 |
| Hap2 | 2 | 0 | 0 | 0 | 0 | 0 | 0 | 0 | 0 | 0 | 0 | 0 | 0 | 0 | 0 | 0 | 0 | 0 | 0 | 0 | 0 | 0 |
| Hap3 | 1 | 0 | 0 | 0 | 0 | 0 | 0 | 0 | 0 | 0 | 0 | 0 | 0 | 0 | 0 | 0 | 0 | 0 | 0 | 0 | 0 | 0 |
| Hap4 | 1 | 0 | 0 | 0 | 0 | 0 | 0 | 0 | 0 | 0 | 0 | 0 | 0 | 0 | 0 | 0 | 0 | 0 | 0 | 0 | 0 | 0 |
| Hap5 | 3 | 0 | 0 | 0 | 0 | 0 | 4 | 10 | 0 | 3 | 3 | 0 | 1 | 0 | 5 | 0 | 0 | 0 | 3 | 0 | 0 | 0 |
| Hap6 | 1 | 0 | 0 | 0 | 0 | 0 | 0 | 0 | 0 | 0 | 0 | 0 | 0 | 0 | 0 | 0 | 0 | 0 | 0 | 0 | 0 | 0 |
| Hap7 | 1 | 0 | 0 | 0 | 0 | 0 | 0 | 0 | 0 | 0 | 0 | 0 | 0 | 0 | 0 | 0 | 0 | 0 | 0 | 0 | 0 | 0 |
| Hap8 | 0 | 1 | 0 | 0 | 0 | 0 | 0 | 0 | 0 | 0 | 0 | 0 | 0 | 0 | 0 | 0 | 0 | 0 | 0 | 0 | 0 | 0 |
| Hap9 | 0 | 1 | 0 | 0 | 0 | 0 | 0 | 0 | 0 | 0 | 0 | 0 | 0 | 0 | 0 | 0 | 0 | 0 | 0 | 0 | 0 | 0 |
| Hap10 | 0 | 1 | 0 | 0 | 0 | 0 | 0 | 0 | 0 | 0 | 0 | 0 | 0 | 0 | 0 | 0 | 0 | 0 | 0 | 0 | 0 | 0 |
| Hap11 | 0 | 1 | 0 | 0 | 0 | 0 | 0 | 0 | 0 | 0 | 0 | 0 | 0 | 0 | 0 | 0 | 0 | 0 | 0 | 0 | 0 | 0 |
| Hap12 | 0 | 0 | 1 | 0 | 0 | 0 | 0 | 0 | 0 | 0 | 0 | 0 | 0 | 0 | 0 | 0 | 0 | 0 | 0 | 0 | 0 | 0 |
| Hap13 | 0 | 0 | 1 | 0 | 0 | 0 | 0 | 0 | 0 | 0 | 0 | 0 | 0 | 0 | 0 | 0 | 0 | 0 | 0 | 0 | 0 | 0 |
| Hap14 | 0 | 0 | 1 | 0 | 0 | 0 | 0 | 0 | 0 | 0 | 0 | 0 | 0 | 0 | 0 | 0 | 0 | 0 | 0 | 0 | 0 | 0 |
| Hap15 | 0 | 0 | 1 | 0 | 0 | 0 | 0 | 0 | 0 | 0 | 0 | 0 | 0 | 0 | 0 | 0 | 0 | 0 | 0 | 0 | 0 | 0 |
| Hap16 | 0 | 0 | 2 | 2 | 0 | 0 | 0 | 0 | 0 | 0 | 0 | 0 | 0 | 0 | 0 | 0 | 0 | 0 | 0 | 0 | 0 | 0 |
| Hap17 | 0 | 0 | 1 | 0 | 0 | 0 | 0 | 0 | 0 | 0 | 0 | 0 | 0 | 0 | 0 | 0 | 0 | 0 | 0 | 0 | 0 | 0 |
| Hap18 | 0 | 0 | 1 | 0 | 0 | 0 | 0 | 0 | 0 | 0 | 0 | 0 | 0 | 0 | 0 | 0 | 0 | 0 | 0 | 0 | 0 | 0 |
| Hap19 | 0 | 0 | 1 | 0 | 0 | 0 | 0 | 0 | 0 | 0 | 0 | 0 | 0 | 0 | 0 | 0 | 0 | 0 | 0 | 0 | 0 | 0 |
| Hap20 | 0 | 0 | 1 | 0 | 0 | 0 | 0 | 0 | 0 | 0 | 0 | 0 | 0 | 0 | 0 | 0 | 0 | 0 | 0 | 0 | 0 | 0 |
| Hap21 | 0 | 0 | 0 | 2 | 0 | 0 | 0 | 0 | 0 | 0 | 0 | 0 | 0 | 0 | 0 | 0 | 0 | 0 | 0 | 0 | 0 | 0 |
| Hap22 | 0 | 0 | 0 | 2 | 0 | 0 | 0 | 0 | 0 | 0 | 0 | 0 | 0 | 0 | 0 | 0 | 0 | 0 | 1 | 0 | 0 | 0 |
| Hap23 | 0 | 0 | 0 | 1 | 0 | 0 | 0 | 0 | 0 | 0 | 0 | 0 | 0 | 0 | 0 | 0 | 0 | 0 | 0 | 0 | 0 | 0 |
| Hap24 | 0 | 0 | 0 | 1 | 0 | 0 | 0 | 0 | 0 | 0 | 0 | 0 | 0 | 0 | 0 | 0 | 0 | 0 | 0 | 0 | 0 | 0 |
| Hap25 | 0 | 0 | 0 | 0 | 1 | 0 | 0 | 0 | 0 | 0 | 0 | 0 | 0 | 0 | 0 | 0 | 0 | 0 | 0 | 0 | 0 | 0 |
| Hap26 | 0 | 0 | 0 | 0 | 0 | 2 | 0 | 0 | 0 | 0 | 0 | 0 | 0 | 0 | 0 | 0 | 0 | 0 | 0 | 0 | 0 | 0 |
| Hap27 | 0 | 0 | 0 | 0 | 0 | 1 | 0 | 0 | 0 | 0 | 0 | 0 | 0 | 0 | 0 | 0 | 0 | 0 | 0 | 0 | 0 | 0 |
| Hap28 | 0 | 0 | 0 | 0 | 0 | 1 | 0 | 0 | 0 | 0 | 1 | 0 | 5 | 6 | 0 | 0 | 0 | 0 | 0 | 0 | 0 | 0 |
| Hap29 | 0 | 0 | 0 | 0 | 0 | 1 | 0 | 0 | 0 | 0 | 0 | 0 | 0 | 0 | 0 | 0 | 0 | 0 | 0 | 0 | 0 | 0 |
| Hap30 | 0 | 0 | 0 | 0 | 0 | 1 | 0 | 0 | 0 | 0 | 0 | 0 | 0 | 0 | 0 | 0 | 0 | 0 | 0 | 0 | 0 | 0 |
| Hap31 | 0 | 0 | 0 | 0 | 0 | 1 | 0 | 0 | 0 | 0 | 0 | 0 | 0 | 0 | 0 | 0 | 0 | 0 | 0 | 0 | 0 | 0 |
| Hap32 | 0 | 0 | 0 | 0 | 0 | 1 | 0 | 0 | 0 | 0 | 0 | 0 | 0 | 0 | 0 | 0 | 0 | 0 | 0 | 0 | 0 | 0 |
| Hap33 | 0 | 0 | 0 | 0 | 0 | 1 | 0 | 0 | 0 | 0 | 0 | 0 | 0 | 0 | 0 | 0 | 0 | 0 | 0 | 0 | 0 | 0 |
| Hap34 | 0 | 0 | 0 | 0 | 0 | 1 | 0 | 0 | 0 | 0 | 0 | 0 | 0 | 0 | 0 | 0 | 0 | 0 | 0 | 0 | 0 | 0 |
| Hap35 | 0 | 0 | 0 | 0 | 0 | 0 | 1 | 0 | 0 | 0 | 0 | 0 | 0 | 0 | 0 | 0 | 0 | 0 | 0 | 0 | 0 | 0 |
| Hap36 | 0 | 0 | 0 | 0 | 0 | 0 | 1 | 0 | 0 | 0 | 0 | 0 | 0 | 0 | 0 | 0 | 0 | 0 | 0 | 0 | 0 | 0 |
| Hap37 | 0 | 0 | 0 | 0 | 0 | 0 | 1 | 0 | 0 | 0 | 0 | 0 | 0 | 0 | 0 | 0 | 0 | 0 | 0 | 0 | 0 | 0 |
| Hap38 | 0 | 0 | 0 | 0 | 0 | 0 | 1 | 0 | 0 | 0 | 0 | 0 | 0 | 0 | 0 | 0 | 0 | 0 | 0 | 0 | 0 | 0 |
| Hap39 | 0 | 0 | 0 | 0 | 0 | 0 | 1 | 0 | 0 | 0 | 0 | 0 | 0 | 0 | 0 | 0 | 0 | 0 | 0 | 0 | 0 | 0 |
| Hap40 | 0 | 0 | 0 | 0 | 0 | 0 | 0 | 0 | 1 | 0 | 0 | 0 | 0 | 0 | 0 | 0 | 0 | 0 | 0 | 0 | 0 | 0 |
| Hap41 | 0 | 0 | 0 | 0 | 0 | 0 | 0 | 0 | 1 | 0 | 0 | 0 | 0 | 0 | 0 | 0 | 0 | 0 | 0 | 0 | 0 | 0 |
| Hap42 | 0 | 0 | 0 | 0 | 0 | 0 | 0 | 0 | 6 | 1 | 0 | 4 | 1 | 0 | 0 | 0 | 0 | 0 | 0 | 0 | 0 | 0 |
| Hap43 | 0 | 0 | 0 | 0 | 0 | 0 | 0 | 0 | 1 | 0 | 0 | 0 | 0 | 0 | 0 | 0 | 0 | 0 | 0 | 0 | 0 | 0 |
| Hap44 | 0 | 0 | 0 | 0 | 0 | 0 | 0 | 0 | 1 | 0 | 1 | 0 | 0 | 0 | 0 | 0 | 0 | 0 | 0 | 0 | 0 | 0 |
| Hap45 | 0 | 0 | 0 | 0 | 0 | 0 | 0 | 0 | 0 | 1 | 0 | 0 | 0 | 0 | 0 | 0 | 0 | 0 | 0 | 0 | 0 | 0 |
| Hap46 | 0 | 0 | 0 | 0 | 0 | 0 | 0 | 0 | 0 | 1 | 0 | 0 | 1 | 0 | 0 | 0 | 0 | 0 | 0 | 0 | 0 | 0 |
| Hap47 | 0 | 0 | 0 | 0 | 0 | 0 | 0 | 0 | 0 | 2 | 0 | 0 | 0 | 0 | 0 | 0 | 0 | 0 | 0 | 0 | 0 | 0 |
| Hap48 | 0 | 0 | 0 | 0 | 0 | 0 | 0 | 0 | 0 | 1 | 0 | 0 | 0 | 0 | 0 | 0 | 0 | 0 | 0 | 0 | 0 | 0 |
| Hap49 | 0 | 0 | 0 | 0 | 0 | 0 | 0 | 0 | 0 | 1 | 0 | 0 | 0 | 0 | 0 | 0 | 0 | 0 | 0 | 0 | 0 | 0 |
| Hap50 | 0 | 0 | 0 | 0 | 0 | 0 | 0 | 0 | 0 | 0 | 1 | 0 | 0 | 0 | 0 | 0 | 0 | 0 | 0 | 1 | 0 | 0 |
| Hap51 | 0 | 0 | 0 | 0 | 0 | 0 | 0 | 0 | 0 | 0 | 1 | 0 | 0 | 0 | 0 | 0 | 0 | 0 | 0 | 0 | 0 | 0 |
| Hap52 | 0 | 0 | 0 | 0 | 0 | 0 | 0 | 0 | 0 | 0 | 1 | 0 | 0 | 0 | 0 | 0 | 0 | 0 | 0 | 0 | 0 | 0 |
| Hap53 | 0 | 0 | 0 | 0 | 0 | 0 | 0 | 0 | 0 | 0 | 1 | 0 | 0 | 0 | 1 | 0 | 0 | 0 | 0 | 0 | 0 | 0 |
| Hap54 | 0 | 0 | 0 | 0 | 0 | 0 | 0 | 0 | 0 | 0 | 0 | 1 | 0 | 0 | 0 | 0 | 0 | 0 | 0 | 0 | 0 | 0 |
| Hap55 | 0 | 0 | 0 | 0 | 0 | 0 | 0 | 0 | 0 | 0 | 0 | 1 | 0 | 0 | 0 | 0 | 0 | 0 | 0 | 0 | 0 | 0 |
| Hap56 | 0 | 0 | 0 | 0 | 0 | 0 | 0 | 0 | 0 | 0 | 0 | 1 | 0 | 0 | 0 | 0 | 0 | 0 | 0 | 0 | 0 | 0 |
| Hap57 | 0 | 0 | 0 | 0 | 0 | 0 | 0 | 0 | 0 | 0 | 0 | 1 | 0 | 0 | 0 | 0 | 0 | 0 | 0 | 0 | 0 | 0 |
| Hap58 | 0 | 0 | 0 | 0 | 0 | 0 | 0 | 0 | 0 | 0 | 0 | 1 | 0 | 0 | 0 | 0 | 0 | 0 | 0 | 0 | 0 | 0 |
| Hap59 | 0 | 0 | 0 | 0 | 0 | 0 | 0 | 0 | 0 | 0 | 0 | 1 | 0 | 0 | 0 | 0 | 0 | 0 | 0 | 0 | 0 | 0 |
| Hap60 | 0 | 0 | 0 | 0 | 0 | 0 | 0 | 0 | 0 | 0 | 0 | 0 | 1 | 0 | 0 | 0 | 0 | 0 | 0 | 0 | 0 | 0 |
| Hap61 | 0 | 0 | 0 | 0 | 0 | 0 | 0 | 0 | 0 | 0 | 0 | 0 | 1 | 0 | 0 | 0 | 0 | 0 | 0 | 0 | 0 | 0 |
| Hap62 | 0 | 0 | 0 | 0 | 0 | 0 | 0 | 0 | 0 | 0 | 0 | 0 | 0 | 1 | 0 | 0 | 0 | 0 | 0 | 0 | 0 | 0 |
| Hap63 | 0 | 0 | 0 | 0 | 0 | 0 | 0 | 0 | 0 | 0 | 0 | 0 | 0 | 1 | 0 | 0 | 0 | 0 | 0 | 0 | 0 | 0 |
| Hap64 | 0 | 0 | 0 | 0 | 0 | 0 | 0 | 0 | 0 | 0 | 0 | 0 | 0 | 2 | 0 | 0 | 0 | 0 | 0 | 0 | 0 | 0 |
| Hap65 | 0 | 0 | 0 | 0 | 0 | 0 | 0 | 0 | 0 | 0 | 0 | 0 | 0 | 0 | 1 | 0 | 0 | 0 | 0 | 0 | 0 | 0 |
| Hap66 | 0 | 0 | 0 | 0 | 0 | 0 | 0 | 0 | 0 | 0 | 0 | 0 | 0 | 0 | 2 | 0 | 0 | 0 | 0 | 0 | 0 | 0 |
| Hap67 | 0 | 0 | 0 | 0 | 0 | 0 | 0 | 0 | 0 | 0 | 0 | 0 | 0 | 0 | 1 | 0 | 0 | 0 | 2 | 0 | 0 | 0 |
| Hap68 | 0 | 0 | 0 | 0 | 0 | 0 | 0 | 0 | 0 | 0 | 0 | 0 | 0 | 0 | 0 | 4 | 0 | 0 | 0 | 0 | 0 | 0 |
| Hap69 | 0 | 0 | 0 | 0 | 0 | 0 | 0 | 0 | 0 | 0 | 0 | 0 | 0 | 0 | 0 | 1 | 0 | 0 | 0 | 0 | 0 | 0 |
| Hap70 | 0 | 0 | 0 | 0 | 0 | 0 | 0 | 0 | 0 | 0 | 0 | 0 | 0 | 0 | 0 | 1 | 0 | 0 | 0 | 0 | 0 | 0 |
| Hap71 | 0 | 0 | 0 | 0 | 0 | 0 | 0 | 0 | 0 | 0 | 0 | 0 | 0 | 0 | 0 | 1 | 0 | 0 | 0 | 0 | 0 | 0 |
| Hap72 | 0 | 0 | 0 | 0 | 0 | 0 | 0 | 0 | 0 | 0 | 0 | 0 | 0 | 0 | 0 | 2 | 1 | 0 | 0 | 0 | 0 | 0 |
| Hap73 | 0 | 0 | 0 | 0 | 0 | 0 | 0 | 0 | 0 | 0 | 0 | 0 | 0 | 0 | 0 | 0 | 3 | 0 | 0 | 0 | 0 | 0 |
| Hap74 | 0 | 0 | 0 | 0 | 0 | 0 | 0 | 0 | 0 | 0 | 0 | 0 | 0 | 0 | 0 | 0 | 3 | 0 | 0 | 0 | 0 | 0 |
| Hap75 | 0 | 0 | 0 | 0 | 0 | 0 | 0 | 0 | 0 | 0 | 0 | 0 | 0 | 0 | 0 | 0 | 2 | 0 | 0 | 0 | 0 | 0 |
| Hap76 | 0 | 0 | 0 | 0 | 0 | 0 | 0 | 0 | 0 | 0 | 0 | 0 | 0 | 0 | 0 | 0 | 1 | 0 | 0 | 0 | 0 | 0 |
| Hap77 | 0 | 0 | 0 | 0 | 0 | 0 | 0 | 0 | 0 | 0 | 0 | 0 | 0 | 0 | 0 | 0 | 0 | 1 | 0 | 0 | 0 | 0 |
| Hap78 | 0 | 0 | 0 | 0 | 0 | 0 | 0 | 0 | 0 | 0 | 0 | 0 | 0 | 0 | 0 | 0 | 0 | 3 | 0 | 0 | 0 | 0 |
| Hap79 | 0 | 0 | 0 | 0 | 0 | 0 | 0 | 0 | 0 | 0 | 0 | 0 | 0 | 0 | 0 | 0 | 0 | 1 | 0 | 0 | 0 | 0 |
| Hap80 | 0 | 0 | 0 | 0 | 0 | 0 | 0 | 0 | 0 | 0 | 0 | 0 | 0 | 0 | 0 | 0 | 0 | 2 | 0 | 0 | 0 | 0 |
| Hap81 | 0 | 0 | 0 | 0 | 0 | 0 | 0 | 0 | 0 | 0 | 0 | 0 | 0 | 0 | 0 | 0 | 0 | 1 | 0 | 0 | 0 | 0 |
| Hap82 | 0 | 0 | 0 | 0 | 0 | 0 | 0 | 0 | 0 | 0 | 0 | 0 | 0 | 0 | 0 | 0 | 0 | 1 | 0 | 0 | 0 | 0 |
| Hap83 | 0 | 0 | 0 | 0 | 0 | 0 | 0 | 0 | 0 | 0 | 0 | 0 | 0 | 0 | 0 | 0 | 0 | 0 | 3 | 0 | 0 | 0 |
| Hap84 | 0 | 0 | 0 | 0 | 0 | 0 | 0 | 0 | 0 | 0 | 0 | 0 | 0 | 0 | 0 | 0 | 0 | 0 | 0 | 3 | 0 | 0 |
| Hap85 | 0 | 0 | 0 | 0 | 0 | 0 | 0 | 0 | 0 | 0 | 0 | 0 | 0 | 0 | 0 | 0 | 0 | 0 | 0 | 1 | 0 | 0 |
| Hap86 | 0 | 0 | 0 | 0 | 0 | 0 | 0 | 0 | 0 | 0 | 0 | 0 | 0 | 0 | 0 | 0 | 0 | 0 | 0 | 1 | 0 | 0 |
| Hap87 | 0 | 0 | 0 | 0 | 0 | 0 | 0 | 0 | 0 | 0 | 0 | 0 | 0 | 0 | 0 | 0 | 0 | 0 | 0 | 1 | 0 | 0 |
| Hap88 | 0 | 0 | 0 | 0 | 0 | 0 | 0 | 0 | 0 | 0 | 0 | 0 | 0 | 0 | 0 | 0 | 0 | 0 | 0 | 1 | 0 | 0 |
| Hap89 | 0 | 0 | 0 | 0 | 0 | 0 | 0 | 0 | 0 | 0 | 0 | 0 | 0 | 0 | 0 | 0 | 0 | 0 | 0 | 1 | 0 | 0 |
| Hap90 | 0 | 0 | 0 | 0 | 0 | 0 | 0 | 0 | 0 | 0 | 0 | 0 | 0 | 0 | 0 | 0 | 0 | 0 | 0 | 1 | 0 | 0 |
| Hap91 | 0 | 0 | 0 | 0 | 0 | 0 | 0 | 0 | 0 | 0 | 0 | 0 | 0 | 0 | 0 | 0 | 0 | 0 | 0 | 0 | 1 | 0 |
| Hap92 | 0 | 0 | 0 | 0 | 0 | 0 | 0 | 0 | 0 | 0 | 0 | 0 | 0 | 0 | 0 | 0 | 0 | 0 | 0 | 0 | 1 | 0 |
| Hap93 | 0 | 0 | 0 | 0 | 0 | 0 | 0 | 0 | 0 | 0 | 0 | 0 | 0 | 0 | 0 | 0 | 0 | 0 | 0 | 0 | 1 | 0 |
| Hap94 | 0 | 0 | 0 | 0 | 0 | 0 | 0 | 0 | 0 | 0 | 0 | 0 | 0 | 0 | 0 | 0 | 0 | 0 | 0 | 0 | 1 | 0 |
| Hap95 | 0 | 0 | 0 | 0 | 0 | 0 | 0 | 0 | 0 | 0 | 0 | 0 | 0 | 0 | 0 | 0 | 0 | 0 | 0 | 0 | 1 | 0 |
| Hap96 | 0 | 0 | 0 | 0 | 0 | 0 | 0 | 0 | 0 | 0 | 0 | 0 | 0 | 0 | 0 | 0 | 0 | 0 | 0 | 0 | 1 | 0 |
| Hap97 | 0 | 0 | 0 | 0 | 0 | 0 | 0 | 0 | 0 | 0 | 0 | 0 | 0 | 0 | 0 | 0 | 0 | 0 | 0 | 0 | 0 | 10 |

**Table S4** Haplotype frequency of the LP lineage.

| Hap | GSLZ | HBSJ | JPIS | JSXZ | KRDJ | LNAS | NXYC | QHXH | SDQD | SXLF | SXTY | SXYA | SXYL |
| --- | --- | --- | --- | --- | --- | --- | --- | --- | --- | --- | --- | --- | --- |
| Hap1 | 6 | 0 | 0 | 0 | 0 | 0 | 0 | 0 | 0 | 0 | 0 | 0 | 1 |
| Hap2 | 1 | 0 | 0 | 0 | 0 | 0 | 0 | 1 | 0 | 0 | 0 | 0 | 0 |
| Hap3 | 1 | 0 | 0 | 0 | 0 | 0 | 0 | 0 | 0 | 0 | 0 | 0 | 0 |
| Hap4 | 1 | 0 | 0 | 0 | 0 | 0 | 0 | 0 | 0 | 0 | 0 | 0 | 0 |
| Hap5 | 1 | 0 | 0 | 0 | 0 | 0 | 0 | 0 | 0 | 0 | 0 | 0 | 0 |
| Hap6 | 0 | 1 | 0 | 0 | 0 | 0 | 0 | 0 | 0 | 0 | 0 | 0 | 0 |
| Hap7 | 0 | 1 | 0 | 0 | 0 | 0 | 0 | 0 | 0 | 0 | 0 | 0 | 0 |
| Hap8 | 0 | 0 | 2 | 0 | 0 | 0 | 0 | 2 | 0 | 0 | 0 | 0 | 0 |
| Hap9 | 0 | 0 | 4 | 0 | 0 | 0 | 0 | 0 | 0 | 0 | 0 | 0 | 0 |
| Hap10 | 0 | 0 | 1 | 0 | 0 | 0 | 0 | 0 | 0 | 0 | 0 | 0 | 0 |
| Hap11 | 0 | 0 | 2 | 0 | 0 | 0 | 0 | 0 | 0 | 0 | 0 | 0 | 0 |
| Hap12 | 0 | 0 | 1 | 0 | 0 | 0 | 0 | 0 | 0 | 0 | 0 | 0 | 0 |
| Hap13 | 0 | 0 | 0 | 1 | 0 | 0 | 0 | 0 | 0 | 0 | 0 | 0 | 0 |
| Hap14 | 0 | 0 | 0 | 0 | 1 | 0 | 0 | 0 | 0 | 0 | 0 | 0 | 0 |
| Hap15 | 0 | 0 | 0 | 0 | 0 | 1 | 0 | 0 | 0 | 0 | 0 | 0 | 0 |
| Hap16 | 0 | 0 | 0 | 0 | 0 | 0 | 1 | 6 | 0 | 0 | 0 | 0 | 0 |
| Hap17 | 0 | 0 | 0 | 0 | 0 | 0 | 0 | 1 | 0 | 0 | 0 | 0 | 0 |
| Hap18 | 0 | 0 | 0 | 0 | 0 | 0 | 0 | 0 | 1 | 1 | 0 | 0 | 0 |
| Hap19 | 0 | 0 | 0 | 0 | 0 | 0 | 0 | 0 | 0 | 2 | 0 | 0 | 0 |
| Hap20 | 0 | 0 | 0 | 0 | 0 | 0 | 0 | 0 | 0 | 1 | 0 | 0 | 0 |
| Hap21 | 0 | 0 | 0 | 0 | 0 | 0 | 0 | 0 | 0 | 1 | 0 | 0 | 0 |
| Hap22 | 0 | 0 | 0 | 0 | 0 | 0 | 0 | 0 | 0 | 1 | 0 | 0 | 0 |
| Hap23 | 0 | 0 | 0 | 0 | 0 | 0 | 0 | 0 | 0 | 2 | 0 | 0 | 0 |
| Hap24 | 0 | 0 | 0 | 0 | 0 | 0 | 0 | 0 | 0 | 1 | 0 | 0 | 0 |
| Hap25 | 0 | 0 | 0 | 0 | 0 | 0 | 0 | 0 | 0 | 1 | 0 | 0 | 0 |
| Hap26 | 0 | 0 | 0 | 0 | 0 | 0 | 0 | 0 | 0 | 0 | 2 | 0 | 0 |
| Hap27 | 0 | 0 | 0 | 0 | 0 | 0 | 0 | 0 | 0 | 0 | 1 | 0 | 0 |
| Hap28 | 0 | 0 | 0 | 0 | 0 | 0 | 0 | 0 | 0 | 0 | 2 | 0 | 0 |
| Hap29 | 0 | 0 | 0 | 0 | 0 | 0 | 0 | 0 | 0 | 0 | 1 | 0 | 0 |
| Hap30 | 0 | 0 | 0 | 0 | 0 | 0 | 0 | 0 | 0 | 0 | 1 | 0 | 0 |
| Hap31 | 0 | 0 | 0 | 0 | 0 | 0 | 0 | 0 | 0 | 0 | 1 | 0 | 0 |
| Hap32 | 0 | 0 | 0 | 0 | 0 | 0 | 0 | 0 | 0 | 0 | 1 | 0 | 0 |
| Hap33 | 0 | 0 | 0 | 0 | 0 | 0 | 0 | 0 | 0 | 0 | 1 | 0 | 0 |
| Hap34 | 0 | 0 | 0 | 0 | 0 | 0 | 0 | 0 | 0 | 0 | 0 | 1 | 0 |
| Hap35 | 0 | 0 | 0 | 0 | 0 | 0 | 0 | 0 | 0 | 0 | 0 | 1 | 0 |
| Hap36 | 0 | 0 | 0 | 0 | 0 | 0 | 0 | 0 | 0 | 0 | 0 | 2 | 0 |
| Hap37 | 0 | 0 | 0 | 0 | 0 | 0 | 0 | 0 | 0 | 0 | 0 | 1 | 0 |
| Hap38 | 0 | 0 | 0 | 0 | 0 | 0 | 0 | 0 | 0 | 0 | 0 | 1 | 0 |
| Hap39 | 0 | 0 | 0 | 0 | 0 | 0 | 0 | 0 | 0 | 0 | 0 | 1 | 0 |
| Hap40 | 0 | 0 | 0 | 0 | 0 | 0 | 0 | 0 | 0 | 0 | 0 | 1 | 0 |
| Hap41 | 0 | 0 | 0 | 0 | 0 | 0 | 0 | 0 | 0 | 0 | 0 | 1 | 0 |
| Hap42 | 0 | 0 | 0 | 0 | 0 | 0 | 0 | 0 | 0 | 0 | 0 | 1 | 0 |
| Hap43 | 0 | 0 | 0 | 0 | 0 | 0 | 0 | 0 | 0 | 0 | 0 | 0 | 1 |
| Hap44 | 0 | 0 | 0 | 0 | 0 | 0 | 0 | 0 | 0 | 0 | 0 | 0 | 1 |
| Hap45 | 0 | 0 | 0 | 0 | 0 | 0 | 0 | 0 | 0 | 0 | 0 | 0 | 1 |
| Hap46 | 0 | 0 | 0 | 0 | 0 | 0 | 0 | 0 | 0 | 0 | 0 | 0 | 1 |
| Hap47 | 0 | 0 | 0 | 0 | 0 | 0 | 0 | 0 | 0 | 0 | 0 | 0 | 1 |
| Hap48 | 0 | 0 | 0 | 0 | 0 | 0 | 0 | 0 | 0 | 0 | 0 | 0 | 1 |
| Hap49 | 0 | 0 | 0 | 0 | 0 | 0 | 0 | 0 | 0 | 0 | 0 | 0 | 1 |

**Table S5** Haplotype frequency of the SH lineage.

| Hap | AHHS | HBWH | HBXY | HNYY | JSNJ | JXZX | SHPT | ZJLS |
| --- | --- | --- | --- | --- | --- | --- | --- | --- |
| Hap1 | 1 | 0 | 0 | 0 | 0 | 0 | 0 | 0 |
| Hap2 | 1 | 0 | 0 | 0 | 0 | 0 | 0 | 0 |
| Hap3 | 1 | 0 | 0 | 0 | 0 | 0 | 0 | 0 |
| Hap4 | 1 | 0 | 0 | 0 | 0 | 0 | 0 | 0 |
| Hap5 | 2 | 0 | 0 | 0 | 0 | 0 | 0 | 0 |
| Hap6 | 0 | 1 | 0 | 0 | 0 | 0 | 0 | 0 |
| Hap7 | 0 | 1 | 0 | 0 | 0 | 0 | 0 | 0 |
| Hap8 | 0 | 1 | 0 | 0 | 0 | 0 | 0 | 0 |
| Hap9 | 0 | 1 | 0 | 0 | 0 | 0 | 0 | 0 |
| Hap10 | 0 | 1 | 0 | 0 | 0 | 0 | 0 | 0 |
| Hap11 | 0 | 2 | 0 | 1 | 0 | 0 | 0 | 0 |
| Hap12 | 0 | 1 | 0 | 0 | 0 | 0 | 0 | 0 |
| Hap13 | 0 | 1 | 0 | 0 | 0 | 0 | 0 | 0 |
| Hap14 | 0 | 0 | 1 | 0 | 0 | 0 | 0 | 0 |
| Hap15 | 0 | 0 | 1 | 0 | 0 | 0 | 0 | 0 |
| Hap16 | 0 | 0 | 1 | 0 | 0 | 0 | 0 | 0 |
| Hap17 | 0 | 0 | 2 | 0 | 0 | 0 | 0 | 0 |
| Hap18 | 0 | 0 | 1 | 0 | 0 | 0 | 0 | 0 |
| Hap19 | 0 | 0 | 1 | 0 | 0 | 0 | 0 | 0 |
| Hap20 | 0 | 0 | 1 | 0 | 0 | 0 | 0 | 0 |
| Hap21 | 0 | 0 | 1 | 0 | 0 | 0 | 0 | 0 |
| Hap22 | 0 | 0 | 1 | 0 | 0 | 0 | 0 | 0 |
| Hap23 | 0 | 0 | 0 | 1 | 0 | 0 | 0 | 0 |
| Hap24 | 0 | 0 | 0 | 1 | 0 | 0 | 0 | 0 |
| Hap25 | 0 | 0 | 0 | 1 | 0 | 0 | 0 | 0 |
| Hap26 | 0 | 0 | 0 | 1 | 0 | 0 | 0 | 0 |
| Hap27 | 0 | 0 | 0 | 1 | 0 | 0 | 0 | 0 |
| Hap28 | 0 | 0 | 0 | 0 | 1 | 0 | 0 | 0 |
| Hap29 | 0 | 0 | 0 | 0 | 3 | 0 | 0 | 0 |
| Hap30 | 0 | 0 | 0 | 0 | 1 | 0 | 0 | 0 |
| Hap31 | 0 | 0 | 0 | 0 | 2 | 0 | 0 | 0 |
| Hap32 | 0 | 0 | 0 | 0 | 1 | 0 | 0 | 0 |
| Hap33 | 0 | 0 | 0 | 0 | 1 | 0 | 0 | 0 |
| Hap34 | 0 | 0 | 0 | 0 | 1 | 0 | 0 | 0 |
| Hap35 | 0 | 0 | 0 | 0 | 0 | 1 | 0 | 0 |
| Hap36 | 0 | 0 | 0 | 0 | 0 | 1 | 0 | 0 |
| Hap37 | 0 | 0 | 0 | 0 | 0 | 1 | 0 | 0 |
| Hap38 | 0 | 0 | 0 | 0 | 0 | 2 | 0 | 0 |
| Hap39 | 0 | 0 | 0 | 0 | 0 | 1 | 0 | 0 |
| Hap40 | 0 | 0 | 0 | 0 | 0 | 1 | 0 | 0 |
| Hap41 | 0 | 0 | 0 | 0 | 0 | 1 | 0 | 0 |
| Hap42 | 0 | 0 | 0 | 0 | 0 | 1 | 0 | 0 |
| Hap43 | 0 | 0 | 0 | 0 | 0 | 0 | 1 | 0 |
| Hap44 | 0 | 0 | 0 | 0 | 0 | 0 | 1 | 0 |
| Hap45 | 0 | 0 | 0 | 0 | 0 | 0 | 1 | 0 |
| Hap46 | 0 | 0 | 0 | 0 | 0 | 0 | 1 | 0 |
| Hap47 | 0 | 0 | 0 | 0 | 0 | 0 | 0 | 2 |
| Hap48 | 0 | 0 | 0 | 0 | 0 | 0 | 0 | 1 |
| Hap49 | 0 | 0 | 0 | 0 | 0 | 0 | 0 | 2 |
| Hap50 | 0 | 0 | 0 | 0 | 0 | 0 | 0 | 1 |
| Hap51 | 0 | 0 | 0 | 0 | 0 | 0 | 0 | 1 |
| Hap52 | 0 | 0 | 0 | 0 | 0 | 0 | 0 | 1 |
| Hap53 | 0 | 0 | 0 | 0 | 0 | 0 | 0 | 1 |
| Hap54 | 0 | 0 | 0 | 0 | 0 | 0 | 0 | 1 |

**Table S6** Mitogenomic coverages and Genbank accession numbers.

| Sample | Reads assembled | Average coverage | Minimum coverage | Maximum coverage | Reference coverage | Genbank accession |
| --- | --- | --- | --- | --- | --- | --- |
| AHHF-1 | 59122 | 552.5 | 116 | 1213 | 100% | MT079333 |
| AHHF-2 | 37337 | 349.6 | 37 | 704 | 100% | MT079334 |
| AHHF-3 | 28204 | 264.1 | 20 | 672 | 100% | MT079335 |
| AHHF-4 | 81796 | 762.3 | 193 | 1678 | 100% | MT079336 |
| AHHF-5 | 82010 | 764.7 | 41 | 1933 | 100% | MT079337 |
| AHHF-6 | 82742 | 772.4 | 110 | 1750 | 100% | MT079338 |
| AHHF-7 | 20244 | 189.6 | 32 | 388 | 100% | MT079339 |
| AHHF-8 | 175758 | 1632.7 | 188 | 3451 | 100% | MT079340 |
| AHHF-9 | 230777 | 2137.3 | 328 | 3696 | 100% | MT079341 |
| AHHF-10 | 231580 | 2232.5 | 330 | 3729 | 100% | MT079342 |
| AHHS-1 | 62391 | 581 | 144 | 1904 | 100% | MT079343 |
| AHHS-2 | 160958 | 1491.2 | 299 | 4140 | 100% | MT079344 |
| AHHS-3 | 90074 | 837.4 | 56 | 2433 | 100% | MT079345 |
| AHHS-4 | 197144 | 1828.2 | 33 | 3903 | 100% | MT079346 |
| AHHS-5 | 24307 | 227.3 | 17 | 639 | 100% | MT079347 |
| AHHS-6 | 68874 | 643 | 107 | 1571 | 100% | MT079348 |
| AHHS-7 | 208247 | 1931.7 | 297 | 3011 | 100% | MT079349 |
| AHHS-8 | 99663 | 928.1 | 62 | 1752 | 100% | MT079350 |
| AHHS-9 | 20541 | 192.4 | 23 | 455 | 100% | MT079351 |
| AHHS-10 | 19980 | 187.2 | 40 | 435 | 100% | MT079352 |
| BJHD-1 | 168999 | 1580.4 | 38 | 4114 | 100% | MT079353 |
| BJHD-2 | 123155 | 1157 | 12 | 3731 | 100% | MT079354 |
| BJHD-3 | 104408 | 972.7 | 39 | 1807 | 100% | MT079355 |
| BJHD-4 | 111929 | 1044.4 | 60 | 1784 | 100% | MT079356 |
| BJHD-5 | 27522 | 258.3 | 10 | 1195 | 100% | MT079357 |
| BJHD-6 | 99315 | 928.2 | 49 | 1604 | 100% | MT079358 |
| BJHD-7 | 43466 | 407.1 | 11 | 1500 | 100% | MT079359 |
| BJHD-8 | 18511 | 173.7 | 12 | 760 | 100% | MT079360 |
| BJHD-9 | 29306 | 274.5 | 13 | 1212 | 100% | MT079361 |
| BJHD-10 | 47669 | 445.4 | 82 | 718 | 100% | MT079362 |
| CQCK-1 | 371909 | 3436.4 | 211 | 8561 | 100% | MT079363 |
| CQCK-2 | 272178 | 2532.7 | 112 | 6631 | 100% | MT079364 |
| CQCK-3 | 254114 | 2363.2 | 54 | 5282 | 100% | MT079365 |
| CQCK-4 | 134190 | 1252.6 | 63 | 2647 | 100% | MT079366 |
| CQCK-5 | 644247 | 5960.8 | 454 | 12449 | 100% | MT079367 |
| CQCK-6 | 78863 | 737.7 | 35 | 1708 | 100% | MT079368 |
| CQCK-7 | 84123 | 785.6 | 89 | 1399 | 100% | MT079369 |
| CQCK-8 | 55318 | 517 | 36 | 1415 | 100% | MT079370 |
| CQCK-9 | 104297 | 973.8 | 109 | 1656 | 100% | MT079371 |
| CQCK-10 | 68201 | 638 | 34 | 1276 | 100% | MT079372 |
| CQJJ-1 | 116621 | 1087.2 | 168 | 4053 | 100% | MT079373 |
| CQJJ-2 | 100038 | 933.2 | 66 | 2657 | 100% | MT079374 |
| CQJJ-3 | 40373 | 377.6 | 64 | 1022 | 100% | MT079375 |
| CQJJ-4 | 35537 | 332.6 | 20 | 1063 | 100% | MT079376 |
| CQJJ-5 | 140005 | 1305.9 | 299 | 3487 | 100% | MT079377 |
| CQJJ-6 | 87104 | 814.2 | 56 | 2070 | 100% | MT079378 |
| CQJJ-7 | 49457 | 462.3 | 95 | 1591 | 100% | MT079379 |
| CQJJ-8 | 3971 | 355.7 | 67 | 882 | 100% | MT079380 |
| CQJJ-9 | 88007 | 822.7 | 152 | 1977 | 100% | MT079381 |
| CQJJ-10 | 36737 | 365.5 | 50 | 970 | 100% | MT079382 |
| GSLZ-1 | 64856 | 606 | 116 | 1097 | 100% | MT079383 |
| GSLZ-2 | 210375 | 1954.4 | 148 | 5050 | 100% | MT079384 |
| GSLZ-3 | 60442 | 564.5 | 66 | 218.7 | 100% | MT079385 |
| GSLZ-4 | 271544 | 2514.8 | 158 | 5677 | 100% | MT079386 |
| GSLZ-5 | 164353 | 1531.2 | 379 | 2910 | 100% | MT079387 |
| GSLZ-6 | 81466 | 761.8 | 40 | 1733 | 100% | MT079388 |
| GSLZ-7 | 112027 | 1044.7 | 50 | 2535 | 100% | MT079389 |
| GSLZ-8 | 79024 | 737.2 | 27 | 1741 | 100% | MT079390 |
| GSLZ-9 | 248219 | 2302.6 | 121 | 5805 | 100% | MT079391 |
| GSLZ-10 | 103015 | 998.5 | 55 | 2449 | 100% | MT079392 |
| GZHZ-1 | 29913 | 279.7 | 30 | 416 | 100% | MT079393 |
| GZHZ-2 | 191096 | 1765.1 | 135 | 2855 | 100% | MT079394 |
| GZHZ-3 | 226318 | 2100.4 | 194 | 2785 | 100% | MT079395 |
| GZHZ-4 | 78932 | 735.2 | 78 | 1032 | 100% | MT079396 |
| GZHZ-5 | 227237 | 2113.5 | 192 | 5151 | 100% | MT079397 |
| GZHZ-6 | 158956 | 1483.3 | 109 | 3253 | 100% | MT079398 |
| GZHZ-7 | 155100 | 1445.4 | 100 | 2978 | 100% | MT079399 |
| GZHZ-8 | 104431 | 973.3 | 60 | 2221 | 100% | MT079400 |
| GZHZ-9 | 153275 | 1428.7 | 96 | 3554 | 100% | MT079401 |
| GZHZ-10 | 116009 | 1081.1 | 70 | 2678 | 100% | MT079402 |
| GZKL-1 | 63547 | 594.2 | 45 | 1972 | 100% | MT079403 |
| GZKL-2 | 85414 | 797.2 | 25 | 2368 | 100% | MT079404 |
| GZKL-3 | 324565 | 3012.4 | 35 | 6343 | 100% | MT079405 |
| GZKL-4 | 121969 | 1137 | 59 | 3405 | 100% | MT079406 |
| GZKL-5 | 150927 | 1406.3 | 100 | 3355 | 100% | MT079407 |
| GZKL-6 | 190524 | 1770.8 | 189 | 4123 | 100% | MT079408 |
| GZKL-7 | 143835 | 1341.6 | 168 | 2945 | 100% | MT079409 |
| GZKL-8 | 118961 | 1109.7 | 54 | 2713 | 100% | MT079410 |
| GZKL-9 | 123646 | 1153.4 | 130 | 2544 | 100% | MT079411 |
| GZKL-10 | 141965 | 1189.5 | 70 | 3508 | 100% | MT079412 |
| HBSJ-1 | 63461 | 593.6 | 33 | 1606 | 100% | MT079413 |
| HBSJ-2 | 105952 | 988.8 | 13 | 3711 | 100% | MT079414 |
| HBSJ-3 | 105138 | 981.2 | 38 | 2334 | 100% | MT079415 |
| HBSJ-4 | 14643 | 137.1 | 18 | 316 | 100% | MT079416 |
| HBSJ-5 | 101265 | 946.1 | 26 | 2420 | 100% | MT079417 |
| HBSJ-6 | 35737 | 334.8 | 20 | 1301 | 100% | MT079418 |
| HBSJ-7 | 104311 | 972.8 | 57 | 2220 | 100% | MT079419 |
| HBSJ-8 | 141756 | 1319.1 | 24 | 3303 | 100% | MT079420 |
| HBSJ-9 | 104336 | 973.4 | 36 | 2924 | 100% | MT079421 |
| HBSJ-10 | 79231 | 741.7 | 19 | 1740 | 100% | MT079422 |
| HBWH-1 | 42212 | 394.4 | 28 | 1080 | 100% | MT079423 |
| HBWH-2 | 113198 | 1056 | 23 | 2516 | 100% | MT079424 |
| HBWH-3 | 40129 | 375.4 | 21 | 1410 | 100% | MT079425 |
| HBWH-4 | 100324 | 935.1 | 29 | 2680 | 100% | MT079426 |
| HBWH-5 | 106250 | 989.3 | 20 | 1604 | 100% | MT079427 |
| HBWH-6 | 126913 | 1183.3 | 293 | 2756 | 100% | MT079428 |
| HBWH-7 | 108290 | 1011.6 | 12 | 2146 | 100% | MT079429 |
| HBWH-8 | 43864 | 410.2 | 13 | 901 | 100% | MT079430 |
| HBWH-9 | 112532 | 1049.8 | 15 | 2388 | 100% | MT079431 |
| HBWH-10 | 102158 | 1000.4 | 29 | 2610 | 100% | MT079432 |
| HBXY-1 | 131239 | 1222.4 | 184 | 2227 | 100% | MT079433 |
| HBXY-2 | 85146 | 794.4 | 99 | 1624 | 100% | MT079434 |
| HBXY-3 | 169903 | 1581.5 | 216 | 2681 | 100% | MT079435 |
| HBXY-4 | 106058 | 992 | 150 | 2192 | 100% | MT079436 |
| HBXY-5 | 34588 | 324.1 | 25 | 1074 | 100% | MT079437 |
| HBXY-6 | 136502 | 1270.6 | 164 | 2635 | 100% | MT079438 |
| HBXY-7 | 102253 | 953.6 | 66 | 2351 | 100% | MT079439 |
| HBXY-8 | 187919 | 1747.2 | 135 | 4065 | 100% | MT079440 |
| HBXY-9 | 116603 | 1086 | 128 | 2260 | 100% | MT079441 |
| HBXY-10 | 252475 | 2343.8 | 20 | 3887 | 100% | MT079442 |
| HNLH-1 | 568096 | 5334.7 | 112 | 18502 | 100% | MT079443 |
| HNLH-2 | 76363 | 716.1 | 30 | 2185 | 100% | MT079444 |
| HNLH-3 | 13464 | 1260.6 | 10 | 3212 | 100% | MT079445 |
| HNLH-4 | 140445 | 1317.1 | 12 | 3216 | 100% | MT079446 |
| HNLH-5 | 174572 | 1626.5 | 15 | 4350 | 100% | MT079447 |
| HNLH-6 | 78952 | 735.6 | 33 | 2265 | 100% | MT079448 |
| HNLH-7 | 100582 | 939 | 14 | 2915 | 100% | MT079449 |
| HNLH-8 | 31225 | 291.7 | 11 | 872 | 100% | MT079450 |
| HNLH-9 | 59547 | 557 | 13 | 1795 | 100% | MT079451 |
| HNLH-10 | 41124 | 321.5 | 20 | 1002 | 100% | MT079452 |
| HNYY-1 | 117159 | 1091.8 | 50 | 3934 | 100% | MT079453 |
| HNYY-2 | 241027 | 2239.9 | 185 | 4958 | 100% | MT079454 |
| HNYY-3 | 60751 | 568.1 | 40 | 1572 | 100% | MT079455 |
| HNYY-4 | 134397 | 1252.4 | 114 | 2838 | 100% | MT079456 |
| HNYY-5 | 157834 | 1470.3 | 123 | 3422 | 100% | MT079457 |
| HNYY-6 | 131022 | 1222.3 | 145 | 2738 | 100% | MT079458 |
| HNYY-7 | 104630 | 976 | 170 | 1748 | 100% | MT079459 |
| HNYY-8 | 44432 | 415.4 | 91 | 848 | 100% | MT079460 |
| HNYY-9 | 88722 | 827.1 | 61 | 2237 | 100% | MT079461 |
| HNYY-10 | 124294 | 1138.5 | 100 | 2840 | 100% | MT079462 |
| JPIS-1 | 156582 | 1452.3 | 332 | 2994 | 100% | MT079463 |
| JPIS-2 | 220257 | 2036.5 | 148 | 4309 | 100% | MT079464 |
| JPIS-3 | 220014 | 2040.5 | 158 | 4115 | 100% | MT079465 |
| JPIS-4 | 236230 | 2192.9 | 356 | 4599 | 100% | MT079466 |
| JPIS-5 | 349011 | 3211.6 | 679 | 7022 | 100% | MT079467 |
| JPIS-6 | 187702 | 1741.1 | 162 | 3840 | 100% | MT079468 |
| JPIS-7 | 182232 | 1683.2 | 253 | 3723 | 100% | MT079469 |
| JPIS-8 | 185560 | 1717.7 | 306 | 2992 | 100% | MT079470 |
| JPIS-9 | 348354 | 3218.1 | 433 | 6281 | 100% | MT079471 |
| JPIS-10 | 185602 | 1731.1 | 159 | 3842 | 100% | MT079472 |
| JSNJ-1 | 75065 | 701.8 | 16 | 2688 | 100% | MT079473 |
| JSNJ-2 | 125880 | 1175.7 | 27 | 2634 | 100% | MT079474 |
| JSNJ-3 | 174194 | 1624.4 | 58 | 3072 | 100% | MT079475 |
| JSNJ-4 | 77742 | 725.9 | 26 | 1785 | 100% | MT079476 |
| JSNJ-5 | 263547 | 2449.9 | 101 | 5233 | 100% | MT079477 |
| JSNJ-6 | 145712 | 1359.5 | 48 | 2684 | 100% | MT079478 |
| JSNJ-7 | 335749 | 3119.1 | 100 | 6708 | 100% | MT079479 |
| JSNJ-8 | 70187 | 655.7 | 27 | 1186 | 100% | MT079480 |
| JSNJ-9 | 285723 | 2659.4 | 42 | 5817 | 100% | MT079481 |
| JSNJ-10 | 306195 | 2829.7 | 100 | 5559 | 100% | MT079482 |
| JSXZ-1 | 113519 | 1056.5 | 114 | 2175 | 100% | MT079483 |
| JSXZ-2 | 105814 | 984.6 | 127 | 1652 | 100% | MT079484 |
| JSXZ-3 | 91550 | 853.4 | 91 | 1787 | 100% | MT079485 |
| JSXZ-4 | 42606 | 398.1 | 26 | 925 | 100% | MT079486 |
| JSXZ-5 | 75912 | 709.8 | 38 | 2061 | 100% | MT079487 |
| JSXZ-6 | 23541 | 220.3 | 22 | 936 | 100% | MT079488 |
| JSXZ-7 | 85852 | 801.7 | 77 | 1432 | 100% | MT079489 |
| JSXZ-8 | 75436 | 705.6 | 59 | 1964 | 100% | MT079490 |
| JSXZ-9 | 40379 | 378 | 25 | 1243 | 100% | MT079491 |
| JSXZ-10 | 42606 | 388.5 | 26 | 934 | 100% | MT079492 |
| JSYN-1 | 59608 | 557.4 | 56 | 2163 | 100% | MT079493 |
| JSYN-2 | 140245 | 1305.5 | 195 | 2208 | 100% | MT079494 |
| JSYN-3 | 111319 | 1035.3 | 132 | 1983 | 100% | MT079495 |
| JSYN-4 | 263230 | 2444.7 | 338 | 4278 | 100% | MT079496 |
| JSYN-5 | 292067 | 2706.2 | 250 | 4889 | 100% | MT079497 |
| JSYN-6 | 172235 | 1601.8 | 297 | 2592 | 100% | MT079498 |
| JSYN-7 | 117001 | 1088.7 | 179 | 1864 | 100% | MT079499 |
| JSYN-8 | 158906 | 1475.8 | 187 | 2384 | 100% | MT079500 |
| JSYN-9 | 138659 | 1289.3 | 183 | 2309 | 100% | MT079501 |
| JSYN-10 | 147824 | 1372 | 130 | 2922 | 100% | MT079502 |
| JXZX-1 | 55406 | 517.3 | 20 | 1218 | 100% | MT079503 |
| JXZX-2 | 45254 | 422.3 | 49 | 938 | 100% | MT079504 |
| JXZX-3 | 104013 | 966.4 | 37 | 2856 | 100% | MT079505 |
| JXZX-4 | 59447 | 555.3 | 25 | 1619 | 100% | MT079506 |
| JXZX-5 | 41957 | 392 | 50 | 938 | 100% | MT079507 |
| JXZX-6 | 52056 | 488.6 | 37 | 1060 | 100% | MT079508 |
| JXZX-7 | 166657 | 1547.5 | 25 | 3200 | 100% | MT079509 |
| JXZX-8 | 140602 | 1308.1 | 123 | 2797 | 100% | MT079510 |
| JXZX-9 | 122930 | 1144.7 | 22 | 2407 | 100% | MT079511 |
| JXZX-10 | 53403 | 520.5 | 20 | 1200 | 100% | MT079512 |
| KRBS-1 | 70675 | 659.3 | 113 | 1266 | 100% | MT079513 |
| KRBS-2 | 68672 | 640.8 | 158 | 983 | 100% | MT079514 |
| KRBS-3 | 40030 | 376 | 32 | 1200 | 100% | MT079515 |
| KRBS-4 | 63611 | 594 | 57 | 981 | 100% | MT079516 |
| KRBS-5 | 108845 | 1015.5 | 223 | 1631 | 100% | MT079517 |
| KRBS-6 | 77998 | 728.7 | 133 | 1243 | 100% | MT079518 |
| KRBS-7 | 183376 | 1708.9 | 343 | 3324 | 100% | MT079519 |
| KRBS-8 | 163193 | 1516.2 | 379 | 3156 | 100% | MT079520 |
| KRBS-9 | 112483 | 1048.6 | 215 | 2146 | 100% | MT079521 |
| KRBS-10 | 247499 | 2291.9 | 493 | 4385 | 100% | MT079522 |
| KRCC-1 | 115307 | 1075.6 | 80 | 2539 | 100% | MT079523 |
| KRCC-2 | 122506 | 1142.3 | 73 | 3021 | 100% | MT079524 |
| KRCC-3 | 168791 | 1571.8 | 91 | 4572 | 100% | MT079525 |
| KRCC-4 | 99439 | 926.8 | 56 | 2378 | 100% | MT079526 |
| KRCC-5 | 167676 | 1560 | 114 | 4021 | 100% | MT079527 |
| KRCC-6 | 190942 | 1773.4 | 133 | 4508 | 100% | MT079528 |
| KRCC-7 | 75610 | 706.2 | 68 | 1236 | 100% | MT079529 |
| KRCC-8 | 148003 | 1380.8 | 123 | 2464 | 100% | MT079530 |
| KRCC-9 | 126095 | 1174.8 | 82 | 2975 | 100% | MT079531 |
| KRCC-10 | 124719 | 1163.1 | 73 | 2882 | 100% | MT079532 |
| KRDJ-1 | 97109 | 906.6 | 69 | 2201 | 100% | MT079533 |
| KRDJ-2 | 115050 | 1073.7 | 55 | 3075 | 100% | MT079534 |
| KRDJ-3 | 84851 | 792.4 | 39 | 2259 | 100% | MT079535 |
| KRDJ-4 | 158798 | 1476.5 | 118 | 3440 | 100% | MT079536 |
| KRDJ-5 | 89822 | 837.2 | 57 | 2026 | 100% | MT079537 |
| KRDJ-6 | 173738 | 1616.5 | 122 | 3750 | 100% | MT079538 |
| KRDJ-7 | 95220 | 888.2 | 59 | 1512 | 100% | MT079539 |
| KRDJ-8 | 108196 | 1008.3 | 60 | 2208 | 100% | MT079540 |
| KRDJ-9 | 131479 | 1224.7 | 99 | 2980 | 100% | MT079541 |
| KRDJ-10 | 15898 | 1452.2 | 94 | 3974 | 100% | MT079542 |
| KRDQ-1 | 165121 | 1535.5 | 108 | 3214 | 100% | MT079543 |
| KRDQ-2 | 197489 | 1848.3 | 544 | 4201 | 100% | MT079544 |
| KRDQ-3 | 20472 | 191.8 | 25 | 617 | 100% | MT079545 |
| KRDQ-4 | 163357 | 1518.5 | 286 | 3331 | 100% | MT079546 |
| KRDQ-5 | 91924 | 857.9 | 94 | 1441 | 100% | MT079547 |
| KRDQ-6 | 51814 | 484.2 | 64 | 860 | 100% | MT079548 |
| KRDQ-7 | 104189 | 973 | 72 | 2245 | 100% | MT079549 |
| KRDQ-8 | 54819 | 512.1 | 23 | 1267 | 100% | MT079550 |
| KRDQ-9 | 108905 | 1017.5 | 40 | 2910 | 100% | MT079551 |
| KRDQ-10 | 129064 | 1204.7 | 97 | 2358 | 100% | MT079552 |
| KRGJ-1 | 64416 | 602.9 | 86 | 1259 | 100% | MT079553 |
| KRGJ-2 | 125556 | 1169.4 | 166 | 2487 | 100% | MT079554 |
| KRGJ-3 | 179179 | 628.6 | 106 | 1237 | 100% | MT079555 |
| KRGJ-4 | 113937 | 1061.2 | 51 | 3140 | 100% | MT079556 |
| KRGJ-5 | 198879 | 1847 | 125 | 2990 | 100% | MT079557 |
| KRGJ-6 | 83989 | 783.2 | 45 | 1748 | 100% | MT079558 |
| KRGJ-7 | 123581 | 1152 | 79 | 2563 | 100% | MT079559 |
| KRGJ-8 | 164491 | 1530.2 | 101 | 3872 | 100% | MT079560 |
| KRGJ-9 | 118599 | 1107.5 | 102 | 1737 | 100% | MT079561 |
| KRGJ-10 | 133964 | 1246.1 | 78 | 3214 | 100% | MT079562 |
| KRSK-1 | 100848 | 941.1 | 20 | 2294 | 100% | MT079563 |
| KRSK-2 | 94095 | 879 | 54 | 1495 | 100% | MT079564 |
| KRSK-3 | 223194 | 2078.1 | 51 | 6176 | 100% | MT079565 |
| KRSK-4 | 129428 | 1205 | 34 | 2569 | 100% | MT079566 |
| KRSK-5 | 189122 | 1761.3 | 140 | 2830 | 100% | MT079567 |
| KRSK-6 | 10783 | 101.2 | 16 | 190 | 100% | MT079568 |
| KRSK-7 | 110262 | 1027.3 | 186 | 1791 | 100% | MT079569 |
| KRSK-8 | 24987 | 233.7 | 19 | 549 | 100% | MT079570 |
| KRSK-9 | 40048 | 373.8 | 35 | 917 | 100% | MT079571 |
| KRSK-10 | 26882 | 251.5 | 14 | 584 | 100% | MT079572 |
| KRSL-1 | 182173 | 1692.9 | 222 | 3294 | 100% | MT079573 |
| KRSL-2 | 91408 | 851.7 | 117 | 1600 | 100% | MT079574 |
| KRSL-3 | 350129 | 3235.3 | 213 | 5498 | 100% | MT079575 |
| KRSL-4 | 203174 | 1884.6 | 138 | 390 | 100% | MT079576 |
| KRSL-5 | 248493 | 2303.1 | 284 | 4402 | 100% | MT079577 |
| KRSL-6 | 175861 | 1640.9 | 186 | 2627 | 100% | MT079578 |
| KRSL-7 | 86593 | 809.7 | 61 | 1873 | 100% | MT079579 |
| KRSL-8 | 238323 | 2219.9 | 300 | 4070 | 100% | MT079580 |
| KRSL-9 | 215751 | 2009.1 | 156 | 3468 | 100% | MT079581 |
| KRSL-10 | 197012 | 1831.8 | 404 | 3494 | 100% | MT079582 |
| LNAS-1 | 57189 | 533.3 | 68 | 1370 | 100% | MT079583 |
| LNAS-2 | 43119 | 402.6 | 15 | 1020 | 100% | MT079584 |
| LNAS-3 | 52705 | 491.9 | 21 | 1270 | 100% | MT079585 |
| LNAS-4 | 35853 | 334.9 | 28 | 925 | 100% | MT079586 |
| LNAS-5 | 42110 | 399.5 | 16 | 1000 | 100% | MT079587 |
| LNAS-6 | 50092 | 467 | 75 | 1199 | 100% | MT079588 |
| LNAS-7 | 22690 | 212.1 | 10 | 687 | 100% | MT079589 |
| LNAS-8 | 59098 | 550.4 | 29 | 1271 | 100% | MT079590 |
| LNAS-9 | 102083 | 950.9 | 54 | 2254 | 100% | MT079591 |
| LNAS-10 | 56767 | 528.8 | 30 | 1272 | 100% | MT079592 |
| LNDL-1 | 16669 | 156.3 | 11 | 452 | 100% | MT079593 |
| LNDL-2 | 38526 | 360.1 | 13 | 1118 | 100% | MT079594 |
| LNDL-3 | 21087 | 197.5 | 29 | 539 | 100% | MT079595 |
| LNDL-4 | 23758 | 222.6 | 10 | 715 | 100% | MT079596 |
| LNDL-5 | 48041 | 449.5 | 11 | 1228 | 100% | MT079597 |
| LNDL-6 | 22052 | 206.5 | 10 | 579 | 100% | MT079598 |
| LNDL-7 | 4830 | 45.3 | 10 | 122 | 100% | MT079599 |
| LNDL-8 | 5627 | 52.8 | 12 | 171 | 100% | MT079600 |
| LNDL-9 | 46393 | 433.7 | 114 | 1071 | 100% | MT079601 |
| LNDL-10 | 21012 | 198 | 12 | 560 | 100% | MT079602 |
| NXYC-1 | 31042 | 290.1 | 20 | 750 | 100% | MT079603 |
| NXYC-2 | 55225 | 514.3 | 68 | 1140 | 100% | MT079604 |
| NXYC-3 | 51331 | 479 | 21 | 1059 | 100% | MT079605 |
| NXYC-4 | 53684 | 501.2 | 68 | 1042 | 100% | MT079606 |
| NXYC-5 | 68864 | 641.9 | 54 | 1626 | 100% | MT079607 |
| NXYC-6 | 47151 | 440 | 67 | 1070 | 100% | MT079608 |
| NXYC-7 | 49634 | 462.7 | 24 | 1279 | 100% | MT079609 |
| NXYC-8 | 30021 | 280.6 | 10 | 702 | 100% | MT079610 |
| NXYC-9 | 64506 | 602.1 | 90 | 1499 | 100% | MT079611 |
| NXYC-10 | 54215 | 510.5 | 55 | 1080 | 100% | MT079612 |
| QHXH-1 | 79890 | 747.3 | 28 | 1614 | 100% | MT079613 |
| QHXH-2 | 109984 | 1028 | 18 | 2014 | 100% | MT079614 |
| QHXH-3 | 163054 | 1521.9 | 49 | 3385 | 100% | MT079615 |
| QHXH-4 | 149676 | 1399.1 | 29 | 3069 | 100% | MT079616 |
| QHXH-5 | 108894 | 1016.5 | 16 | 1876 | 100% | MT079617 |
| QHXH-6 | 157417 | 1470.7 | 23 | 2968 | 100% | MT079618 |
| QHXH-7 | 84002 | 785.3 | 67 | 1579 | 100% | MT079619 |
| QHXH-8 | 108416 | 1013.5 | 59 | 2367 | 100% | MT079620 |
| QHXH-9 | 135398 | 1264.7 | 22 | 2444 | 100% | MT079621 |
| QHXH-10 | 134265 | 1129.5 | 23 | 2420 | 100% | MT079622 |
| SCAB-1 | 141669 | 1318.1 | 84 | 3534 | 100% | MT079623 |
| SCAB-2 | 65640 | 611.7 | 44 | 1392 | 100% | MT079624 |
| SCAB-3 | 90155 | 840.6 | 86 | 1832 | 100% | MT079625 |
| SCAB-4 | 140545 | 1306.6 | 208 | 2817 | 100% | MT079626 |
| SCAB-5 | 274699 | 2557.7 | 81 | 5282 | 100% | MT079627 |
| SCAB-6 | 156169 | 1456.6 | 294 | 2647 | 100% | MT079628 |
| SCAB-7 | 178809 | 1667.9 | 63 | 3492 | 100% | MT079629 |
| SCAB-8 | 542872 | 5027.9 | 372 | 9417 | 100% | MT079630 |
| SCAB-9 | 215587 | 2005.8 | 378 | 4909 | 100% | MT079631 |
| SCAB-10 | 155165 | 1462.3 | 290 | 2730 | 100% | MT079632 |
| SDQD-1 | 149854 | 1397 | 68 | 3053 | 100% | MT079633 |
| SDQD-2 | 14868 | 139.3 | 20 | 323 | 100% | MT079634 |
| SDQD-3 | 7980 | 74.9 | 10 | 241 | 100% | MT079635 |
| SDQD-4 | 56816 | 531.5 | 28 | 1137 | 100% | MT079636 |
| SDQD-5 | 52327 | 488.5 | 123 | 1062 | 100% | MT079637 |
| SDQD-6 | 5727 | 53.8 | 10 | 142 | 100% | MT079638 |
| SDQD-7 | 93319 | 872.5 | 32 | 2289 | 100% | MT079639 |
| SDQD-8 | 38216 | 357.7 | 57 | 973 | 100% | MT079640 |
| SDQD-9 | 37544 | 351.9 | 15 | 1197 | 100% | MT079641 |
| SDQD-10 | 23652 | 221.5 | 16 | 422 | 100% | MT079642 |
| SDTA-1 | 207188 | 1929.5 | 108 | 4668 | 100% | MT079643 |
| SDTA-2 | 159940 | 1488 | 63 | 3217 | 100% | MT079644 |
| SDTA-3 | 69070 | 644.2 | 48 | 1649 | 100% | MT079645 |
| SDTA-4 | 56645 | 529.9 | 17 | 1676 | 100% | MT079646 |
| SDTA-5 | 61917 | 580.1 | 16 | 1862 | 100% | MT079647 |
| SDTA-6 | 13768 | 129 | 10 | 615 | 100% | MT079648 |
| SDTA-7 | 22613 | 212.3 | 10 | 946 | 100% | MT079649 |
| SDTA-8 | 37236 | 348.8 | 14 | 1370 | 100% | MT079650 |
| SDTA-9 | 102151 | 954.1 | 15 | 3618 | 100% | MT079651 |
| SDTA-10 | 59402 | 554.3 | 151 | 848 | 100% | MT079652 |
| SHPT-1 | 64903 | 606.2 | 30 | 1215 | 100% | MT079653 |
| SHPT-2 | 149148 | 1387.8 | 21 | 3039 | 100% | MT079654 |
| SHPT-3 | 70296 | 656.1 | 15 | 1600 | 100% | MT079655 |
| SHPT-4 | 46840 | 438.1 | 26 | 1950 | 100% | MT079656 |
| SHPT-5 | 31641 | 296.2 | 22 | 1059 | 100% | MT079657 |
| SHPT-6 | 31040 | 290.4 | 13 | 1500 | 100% | MT079658 |
| SHPT-7 | 21431 | 197.6 | 24 | 978 | 100% | MT079659 |
| SHPT-8 | 103691 | 968.3 | 93 | 2940 | 100% | MT079660 |
| SHPT-9 | 16818 | 157.5 | 23 | 648 | 100% | MT079661 |
| SHPT-10 | 32341 | 285.3 | 20 | 1489 | 100% | MT079662 |
| SXLF-1 | 253862 | 2370.8 | 57 | 4763 | 100% | MT079663 |
| SXLF-2 | 130645 | 1221.5 | 18 | 814.8 | 100% | MT079664 |
| SXLF-3 | 319093 | 2979.2 | 64 | 7042 | 100% | MT079665 |
| SXLF-4 | 102188 | 954.5 | 44 | 2063 | 100% | MT079666 |
| SXLF-5 | 97721 | 913.7 | 30 | 1893 | 100% | MT079667 |
| SXLF-6 | 200231 | 1869.5 | 28 | 4842 | 100% | MT079668 |
| SXLF-7 | 72350 | 677.8 | 18 | 1700 | 100% | MT079669 |
| SXLF-8 | 83225 | 778.7 | 16 | 1761 | 100% | MT079670 |
| SXLF-9 | 104046 | 972.2 | 26 | 2351 | 100% | MT079671 |
| SXLF-10 | 293330 | 2733.5 | 35 | 6181 | 100% | MT079672 |
| SXTY-1 | 93517 | 873.1 | 10 | 2210 | 100% | MT079673 |
| SXTY-2 | 124030 | 1158.3 | 16 | 3055 | 100% | MT079674 |
| SXTY-3 | 7122 | 66.5 | 10 | 420 | 100% | MT079675 |
| SXTY-4 | 28851 | 269.7 | 12 | 920 | 100% | MT079676 |
| SXTY-5 | 21470 | 201.3 | 11 | 863 | 100% | MT079677 |
| SXTY-6 | 91026 | 850.1 | 21 | 2643 | 100% | MT079678 |
| SXTY-7 | 17145 | 160.5 | 11 | 633 | 100% | MT079679 |
| SXTY-8 | 40472 | 378 | 21 | 1154 | 100% | MT079680 |
| SXTY-9 | 16630 | 155 | 10 | 1100 | 100% | MT079681 |
| SXTY-10 | 17145 | 158.5 | 14 | 639 | 100% | MT079682 |
| SXYA-1 | 74330 | 692.2 | 34 | 1754 | 100% | MT079683 |
| SXYA-2 | 91587 | 853.4 | 56 | 1626 | 100% | MT079684 |
| SXYA-3 | 80112 | 747.6 | 36 | 1745 | 100% | MT079685 |
| SXYA-4 | 91750 | 855.3 | 28 | 2335 | 100% | MT079686 |
| SXYA-5 | 37196 | 347.8 | 10 | 776 | 100% | MT079687 |
| SXYA-6 | 53783 | 502.5 | 11 | 1258 | 100% | MT079688 |
| SXYA-7 | 44697 | 417.2 | 58 | 1120 | 100% | MT079689 |
| SXYA-8 | 52937 | 494.2 | 21 | 1275 | 100% | MT079690 |
| SXYA-9 | 66814 | 623.5 | 26 | 1590 | 100% | MT079691 |
| SXYA-10 | 51735 | 489.4 | 17 | 1260 | 100% | MT079692 |
| SXYL-1 | 15511 | 145.3 | 11 | 451 | 100% | MT079693 |
| SXYL-2 | 43612 | 407.3 | 14 | 1086 | 100% | MT079694 |
| SXYL-3 | 83059 | 771.2 | 34 | 2062 | 100% | MT079695 |
| SXYL-4 | 21438 | 200.7 | 10 | 615 | 100% | MT079696 |
| SXYL-5 | 173934 | 1610.4 | 115 | 3284 | 100% | MT079697 |
| SXYL-6 | 142252 | 1317 | 72 | 2329 | 100% | MT079698 |
| SXYL-7 | 129086 | 1198.3 | 76 | 3452 | 100% | MT079699 |
| SXYL-8 | 124780 | 1155.1 | 43 | 3211 | 100% | MT079700 |
| SXYL-9 | 76458 | 707.6 | 14 | 2246 | 100% | MT079701 |
| SXYL-10 | 124780 | 1149.6 | 36 | 3100 | 100% | MT079702 |
| USPA-1 | 30264 | 276.5 | 20 | 688 | 100% | MT079703 |
| USPA-2 | 28208 | 264.1 | 10 | 832 | 100% | MT079704 |
| USPA-3 | 30264 | 282.9 | 11 | 693 | 100% | MT079705 |
| USPA-4 | 34975 | 326.6 | 49 | 684 | 100% | MT079706 |
| USPA-5 | 89919 | 837.2 | 91 | 1333 | 100% | MT079707 |
| USPA-6 | 79549 | 741.2 | 42 | 1206 | 100% | MT079708 |
| USPA-7 | 17980 | 169.4 | 10 | 528 | 100% | MT079709 |
| USPA-8 | 19443 | 182 | 12 | 564 | 100% | MT079710 |
| USPA-9 | 37881 | 354 | 10 | 988 | 100% | MT079711 |
| USPA-10 | 19627 | 184 | 11 | 571 | 100% | MT079712 |
| YNPB-1 | 122268 | 1139.8 | 126 | 1883 | 100% | MT079713 |
| YNPB-2 | 126801 | 1181.8 | 145 | 1884 | 100% | MT079714 |
| ZJLS-1 | 105852 | 983.1 | 179 | 2171 | 100% | MT079715 |
| ZJLS-2 | 124335 | 1156.5 | 182 | 2592 | 100% | MT079716 |
| ZJLS-3 | 128000 | 1188.7 | 199 | 2704 | 100% | MT079717 |
| ZJLS-4 | 43679 | 407.7 | 48 | 1121 | 100% | MT079718 |
| ZJLS-5 | 236907 | 2190.8 | 338 | 965.5 | 100% | MT079719 |
| ZJLS-6 | 111581 | 1041.4 | 109 | 2237 | 100% | MT079720 |
| ZJLS-7 | 136918 | 1275.4 | 138 | 2591 | 100% | MT079721 |
| ZJLS-8 | 52887 | 494.8 | 56 | 1166 | 100% | MT079722 |
| ZJLS-9 | 57895 | 541.6 | 44 | 1565 | 100% | MT079723 |
| ZJLS-10 | 128000 | 1201.4 | 200 | 2621 | 100% | MT079724 |
| *Lycorma meliae* | 322066 | 2976.6 | 347 | 6338 | 100% | MT079725 |
